# Multiscale Profiling of Nanoscale Metal‐Organic Framework Biocompatibility and Immune Interactions

**DOI:** 10.1002/adhm.202501809

**Published:** 2025-08-07

**Authors:** Yunhui Zhuang, Bárbara B. Mendes, Dhruv Menon, Jhenifer Oliveira, Xu Chen, Fatma Demir Duman, João Conniot, Sergio Mercado, Xiewen Liu, Shi‐Yuan Zhang, João Conde, Rachel E. Hewitt, David Fairen‐Jimenez

**Affiliations:** ^1^ Adsorption & Advanced Materials Laboratory (AAML) Department of Chemical Engineering and Biotechnology University of Cambridge Philippa Fawcett Drive Cambridge CB3 0AS UK; ^2^ Comprehensive Health Research Centre (CHRC), NOVA Medical School, Faculdade de Ciências Médicas NMS FCM Universidade NOVA de Lisboa Lisbon Portugal; ^3^ Cellular Imaging and Analysis Facility Department of Veterinary Medicine University of Cambridge Madingley Road Cambridge CB3 0ES UK

**Keywords:** cytokines, immunotoxicity, metal‐organic frameworks (MOFs), monocytes, machine learning

## Abstract

The clinical translation of metal‐organic frameworks (MOFs) – a promising class of porous materials for nanomedicine – is hindered by a poor understanding of their complex interactions with the immune system and in vivo immunotoxicity. To address this gap, a hierarchical “Safety‐by‐Design” pipeline is established and validated, integrating machine learning (ML) with ex vivo human blood studies and targeted in vivo models. This multi‐stage workflow enables the systematic profiling of MOF immunotoxicity, de‐risking their development. The power of this approach is demonstrated using four clinically relevant MOFs – NU‐901, PCN‐222, UiO‐66, and ZIF‐8 – revealing distinct, framework‐dependent immune fingerprints. The initial in silico screening correctly flagged NU‐901 and ZIF‐8 as potential hazards to human health. These predictions are subsequently validated ex vivo, where NU‐901 is confirmed to be selectively cytotoxic to CD14^+^ monocytes, and ZIF‐8 is identified as a specific pro‐inflammatory agent via IL‐6 induction. In contrast, candidates predicted to be safe – UiO‐66 and PCN‐222 – demonstrated high biocompatibility ex vivo and advanced to in vivo studies, where they caused only minimal and transient immune activation. This study provides a validated, resource‐efficient roadmap for preclinical immunotoxicity assessment, establishing a rational paradigm to accelerate the safe clinical translation of MOFs and other advanced nanomedicines.

## Graphical abstract

A multi‐scale, hierarchical ‘Safety‐by‐Design’ pipeline combining machine learning, ex vivo human blood assays, and in vivo models enables the systematic immunotoxicity and biocompatibility profiling of nanoscale metal‐organic frameworks, accelerating their safe clinical translation.

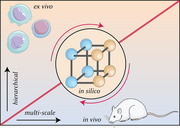

## 1 Introduction

Nanomedicine enables the transport and delivery of active pharmaceutical ingredients (APIs) while regulating their release kinetics, resulting in optimal pharmacokinetics. This makes it a promising strategy for targeted drug delivery, bioimaging, and diagnostics.^[^ ^1^, ^2^ ^]^ Within the broad spectrum of nanomaterials, metal‐organic frameworks (MOFs) are being extensively studied for such applications due to their high porosity, large surface area, and easily modifiable external surface functionalities.^[^ ^3^, ^4^, ^5^ ^]^ Pioneering work from Horcajada, Gref, Lin, Serre, and others have driven MOFs into the spotlight as viable nanocarriers for drug delivery.^[^ ^3^, ^4^, ^6^, ^7^ ^]^ MOFs are highly effective for delivering hydrophobic and hydrophilic drugs, enhancing solubility, release profiles, and drug half‐life. Furthermore, they can transport macromolecules such as peptides and nucleic acids, protecting them from enzymatic degradation.^[^ ^8^, ^9^, ^10^ ^]^

Among reported MOFs, Zn‐, Fe‐, and Zr‐based frameworks have garnered substantial attention for biomedical applications due to their favorable biocompatibility and stability profiles. These materials strike a critical balance – wherein they must remain stable enough to effectively deliver the API, while being degradable to prevent bioaccumulation.^[^ ^11^ ^]^ The chemical versatility of MOFs enables post‐synthetic modifications, allowing precise functionalization.^[^ ^1^, ^12^ ^]^ We have demonstrated the use of Zr‐MOFs for targeted drug delivery to both the cytosol and mitochondria.^[^ ^13^, ^14^ ^]^ By tailoring particle size and surface chemistry, we and others have directed cellular uptake via endocytosis, emphasizing the crucial role of size in cellular uptake and cytotoxicity.^[^ ^4^, ^15^ ^]^ Our previous studies have investigated how nanoparticle properties – such as metal composition, topology, solubility, and surface chemistry – affect cellular interactions in vitro and in vivo.^[^ ^1^, ^15^ ^]^ Recently, we showed that PEGylation and bilayer modifications of Zr‐MOFs (UiO‐66, NU‐901, PCN‐128, and PCN‐222) significantly enhance colloidal stability and dispersity in biological solvents.^[^ ^16^, ^17^ ^]^

Despite significant progress in biomedical applications, comprehensive profiling of MOF nanotoxicology remains limited, posing a challenge for clinical translation.^[^ ^18^, ^19^ ^]^ Key barriers include limited understanding of nanoparticle interactions with biological systems and a lack of standardization in biological assays.^[^ ^20^ ^]^ In vitro cytotoxicity evaluations typically rely on metabolic‐based assays (e.g., MTT, Alamar Blue) and cell proliferation studies, which, while high‐throughput, do not sufficiently capture MOF interactions with cells and their microenvironment under clinically relevant conditions.^[^ ^19^, ^20^, ^21^ ^]^ Effective nanotoxicity examinations must consider the route of administration and initial interactions with biological components.^[^ ^15^, ^19^ ^]^ Intravenous (IV) injection is the preferred method for in vivo studies because it bypasses gastrointestinal barriers, enhances bioavailability, and maximize biodistribution.^[^ ^22^, ^23^ ^]^ However, IV delivery also introduces risks such as burst release, leading to rapid nanoparticle accumulation in systemic circulation and potential side effects.

Following administration, MOFs interact with the immune system, potentially triggering immunosuppression, immunostimulation, hypersensitivity, or autoimmunity.^[^ ^24^, ^25^ ^]^ Evaluating their immunotoxicity is, thus, essential for safe clinical translation.^[^ ^18^, ^25^ ^]^ However, differences between human and animal immune responses pose significant challenges in predicting clinical outcomes. While in vitro and in vivo studies using murine macrophages, zebrafish embryos, and mouse models provide important insights, they are unable to capture the complexities of MOF immunotoxicology.^[^ ^26^, ^27^, ^28^ ^]^ Human immunity involves complex interactions between soluble inflammatory mediators (e.g., cytokines) and various cell‐cell dynamics between tissues.^[^ ^29^, ^30^ ^]^ To address these limitations, in silico studies have emerged as a key tool for early‐stage screening.^[^ ^31^ ^]^ For example, we have reported computational methods to predict MOF interactions with biological systems, offering preliminary assessments of toxicity.^[^ ^32^ ^]^ These models are cost‐ and resource‐effective and time‐saving, enabling us to identify promising candidates while reducing the risk of late‐stage failures.^[^ ^26^, ^32^ ^]^

Ex vivo studies using human peripheral blood mononuclear cells (PBMCs) further bridge the gap between in vitro models and their clinical applicability. PBMCs – a mixed population of immune cells, including T and B lymphocytes, monocytes, natural killer cells, and dendritic cells – are widely used to model immune responses in vaccine development and cancer research. They also serve as a valuable tool for assessing nanoparticle‐induced immune responses and cytotoxicity.^[^ ^33^ ^]^ Functional evaluation of immune cells exposed to MOFs is important to identify adverse reactions such as lymphocyte activation or cytokine production, which may increase risks of infection, cancer, or autoimmune diseases.^[^ ^24^, ^30^, ^34^ ^]^ In vivo studies remain indispensable for evaluating the full biological impact of MOFs across different administration routes.^[^ ^35^ ^]^ They provide insights into biocompatibility, toxicity, inflammation, and organ damage while validating findings from in silico, in vitro, and ex vivo models – ensuring MOFs are safe and effective before progressing to potential clinical trials.

In this study, we have established and validated a multi‐scale profiling pipeline as a framework to address the immunotoxicity challenges hindering the clinical translation of nanoscale MOFs (_nano_MOFs). Our comprehensive approach begins with in silico machine learning (ML)‐based toxicity screening, which guides a detailed ex vivo investigation using human peripheral blood mononuclear cells (PBMCs). In this second stage, we assess cell population dynamics, the viability of CD14^+^ monocytes and CD3^+^ T lymphocytes, and quantify cellular interactions using imaging flow cytometry and inflammatory cytokine arrays. Finally, the most promising candidates were advanced to in vivo mouse models for systemic evaluation. The goal of this integrated pipeline is to generate a nuanced “immune fingerprint” for each material, allowing us to distinguish between different modes of immune interaction. By applying this framework, we aim to specifically identify and prioritize candidates suitable for passive drug delivery applications – where minimal immunogenicity is the paramount design criterion. This methodology provides a rational, tiered approach to inform safety profiles and accelerate the clinical translation of MOF‐based nanomedicines.

## 2 In silico Assessment of MOFs

**Figure 1:**
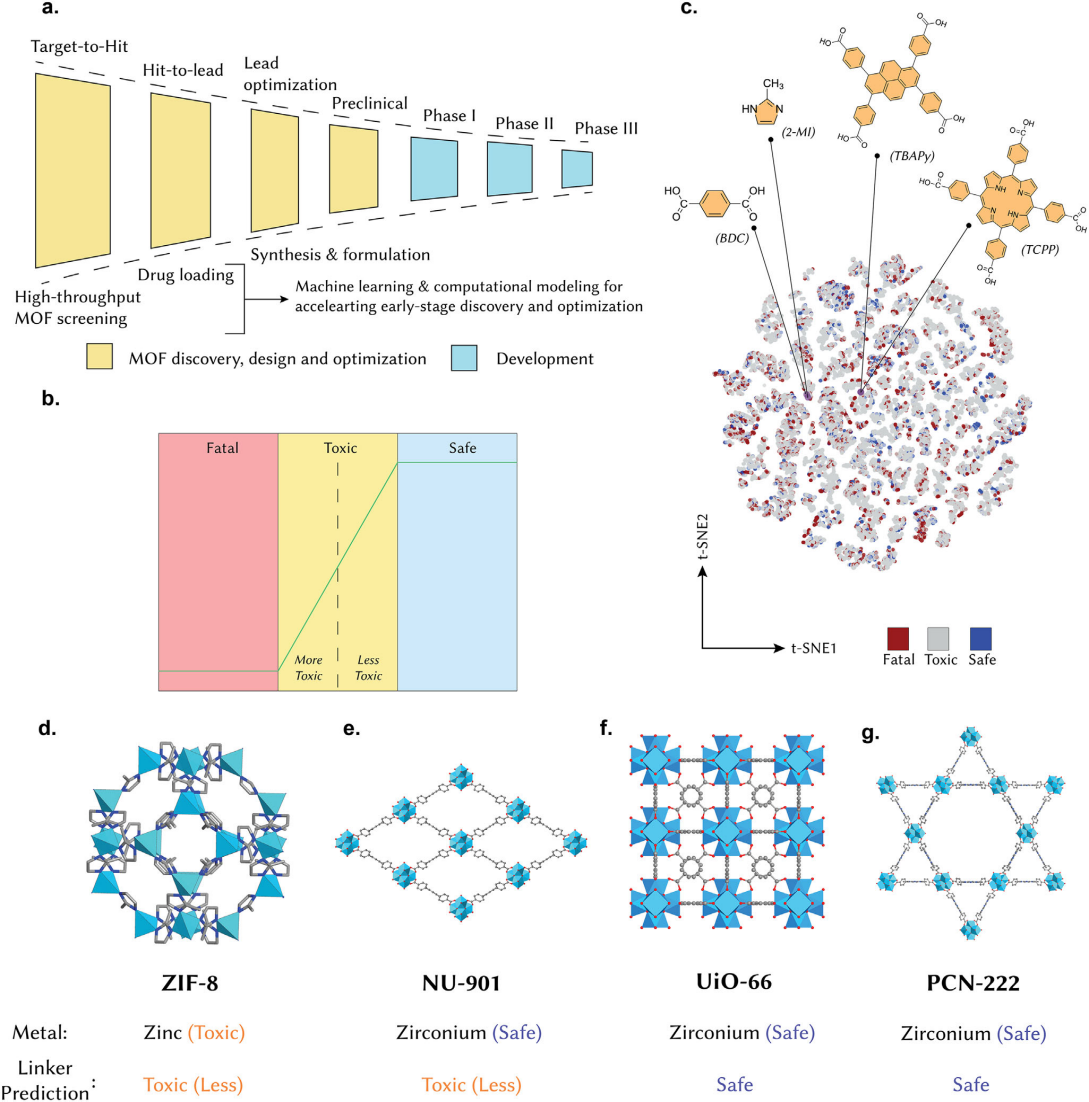
In silico screening of MOFs. a) The conventional drug discovery and development pipeline adapted to the clinical translation of MOFs. The conventional pipeline presented here has been inspired from Paul et al.^[^ ^37^ ^]^ b) The classification criteria developed for predicting the toxicity profiles of MOF linkers. Thresholds are based on the UN GHS.^[^ ^39^ ^]^ For a detailed description of the ML models, we refer the reader to our previous work.^[^ ^32^ ^]^ c) The chemical landscape of 35 047 organic molecules is color‐coded according to their toxicity classification. Points in purple correspond to the MOF linkers of interest. The landscape was generated using t‐distributed stochastic neighborhood embedding (tSNE). Toxicity classification of the MOFs investigated in this study: d) ZIF‐8, e) NU‐901, f) UiO‐66, and g) PCN‐222. Linker toxicity corresponds to intraperitoneal route of administration.

The clinical translation of new molecular entities (NMEs) faces persistent challenges from high US FDA attrition rates, with mean development costs reaching $1.5 billion (2018 estimates).^[^ ^36^, ^37^ ^]^ Although clinical success rates have improved post‐2014,^[^ ^38^ ^]^ in their landmark analysis, Paul et al.^[^ ^37^ ^]^ emphasized that without a “dramatic” increase in R&D productivity, pharmaceutical innovation would remain unsustainable. Reducing Phase II/III attrition through strengthened (and accelerated) early‐stage development is critical (**Figure** **1**a), as, on average, for every 9 NMEs that enter clinical development, typically, only one receives FDA approval.

For MOFs, several of these hurdles may intensify due to: i. increased regulatory scrutiny, as MOF‐based formulations may fall under the “truly innovative new medicine” category, and ii. vast precursor diversity which complicates experimental discovery and design optimization.^[^ ^26^, ^32^ ^]^ To address some of these challenges, we developed a ML‐guided computational pipeline for the high‐throughput screening of biocompatibility of MOFs based on precursor toxicity.^[^ ^32^ ^]^ For the linker, we developed hierarchical models that first classify linkers as “fatal,” “toxic,” or “safe” based on median lethal dose (LD_50_) thresholds in mice when administered using the intraperitoneal and oral routes, then refine toxicity grades for “toxic” candidates (Figure 1b). Figure 1c shows the chemical landscape of 35,047 organic molecules, color‐coded according to their toxicity classification when administered via the intraperitoneal route. The toxicity classifications were based on the United Nations “Globally Harmonized System of the Classification and Labelling of Chemicals” (UN GHS).^[^ ^39^ ^]^ This enables the high‐throughput, “safety‐by‐design” (SbD)^[^ ^40^ ^]^ screening of MOF libraries to prioritize biocompatible frameworks, and extract design trends.^[^ ^32^ ^]^

While our initial studies focused on the high‐throughput screening of large MOF databases to choose optimal, biocompatible candidates for drug delivery,^[^ ^26^, ^32^ ^]^ here, we sought to progress one step further by bridging in silico analysis with mechanistic, experimental validation. In doing so, we deliberated on the selection of a varied cohort of materials to potentially study the fundamental structure‐property relationships that govern immunotoxicity. Thus, based on the historical context from the preceding literature^[^ ^4^, ^35^, ^41^, ^42^ ^]^ and our current expertise,^[^ ^16^, ^17^, ^26^, ^32^ ^]^ we chose four specific and potentially clinically relevant MOFs for our investigation – ZIF‐8, NU‐901, UiO‐66, and PCN‐222. The chosen MOFs represent a diverse set of chemical and structural features that may inspire future design trends. With regards to the metal center, Zn and Zr represent two of the most common metals used in biomedical applications of MOFs. Our selection features two microporous (ZIF‐8, UiO‐66) and two mesoporous (NU‐901, PCN‐222) MOFs, which influence the drug loading capacity and release kinetics. Furthermore, the diverse array of linkers includes a simple carboxylate (in UiO‐66), an imidazole derivative (in ZIF‐8), a complex porphyrin (in PCN‐222), and a polycyclic aromatic hydrocarbon (PAH) derivative (in NU‐901), thereby spanning a considerably diverse chemical space.

When applied to ZIF‐8, NU‐901, UiO‐66, and PCN‐222, the pipeline yielded predictions that provided immediate mechanistic hypotheses. For ZIF‐8, both the Zn metal center and 2‐methylimidazole linker were classified as “toxic” (Figure 1d). NU‐901 showed a mixed profile, with a “safe” Zr center and a “toxic” pyrene linker (Figure 1e). UiO‐66 was predicted to be “safe” for both the Zr center and carboxylic linker (Figure 1f). Similarly, PCN‐222 received a “safe” classification for both the Zr center and porphyrin linker (Figure 1g).

Our model's flagging of the 1,3,6,8‐tetrakis(p‐benzoic acid) pyrene (H_4_TBAPy) linker in NU‐901 is mechanistically insightful. The linker is a derivative of pyrene – a PAH – i.e., a class of compounds well documented for their immunotoxicity, including the suppression of immune cell function and the induction of apoptosis in macrophages and lymphocytes.^[^ ^43^, ^44^ ^]^ The model, therefore, correctly identified a structural motif with an established history of adverse biological interactions. Similarly, the “toxic” prediction for ZIF‐8 is theoretically reasonable. ZIF‐8 is known to be labile in acidic environments^[^ ^45^ ^]^ – such as those in phagocytic endosomes – where it degrades and releases its linker. The imidazole moiety in particular is a known structural feature of agonists for endosomal Toll‐like receptors (TLRs) – particularly TLR‐7.^[^ ^46^, ^47^, ^48^ ^]^ Agonistic activation of these receptors triggers downstream inflammatory signaling pathways that lead to the production of cytokines.^[^ ^46^ ^]^

The ability of our pipeline to generate testable, mechanism‐based hypotheses is a core component of the SbD approach. While the model points toward likely toxicity mechanisms and identifies mitigation strategies such as bioisostere substitutions and linker functionalization, it cannot replace experimental validation.^[^ ^32^ ^]^ Therefore, in the subsequent sections, we bridge mechanistic gaps by experimentally validating these computational predictions through detailed immune profiling and biodistribution studies.

## 3 MOF Synthesis and Formulation

**Figure 2:**
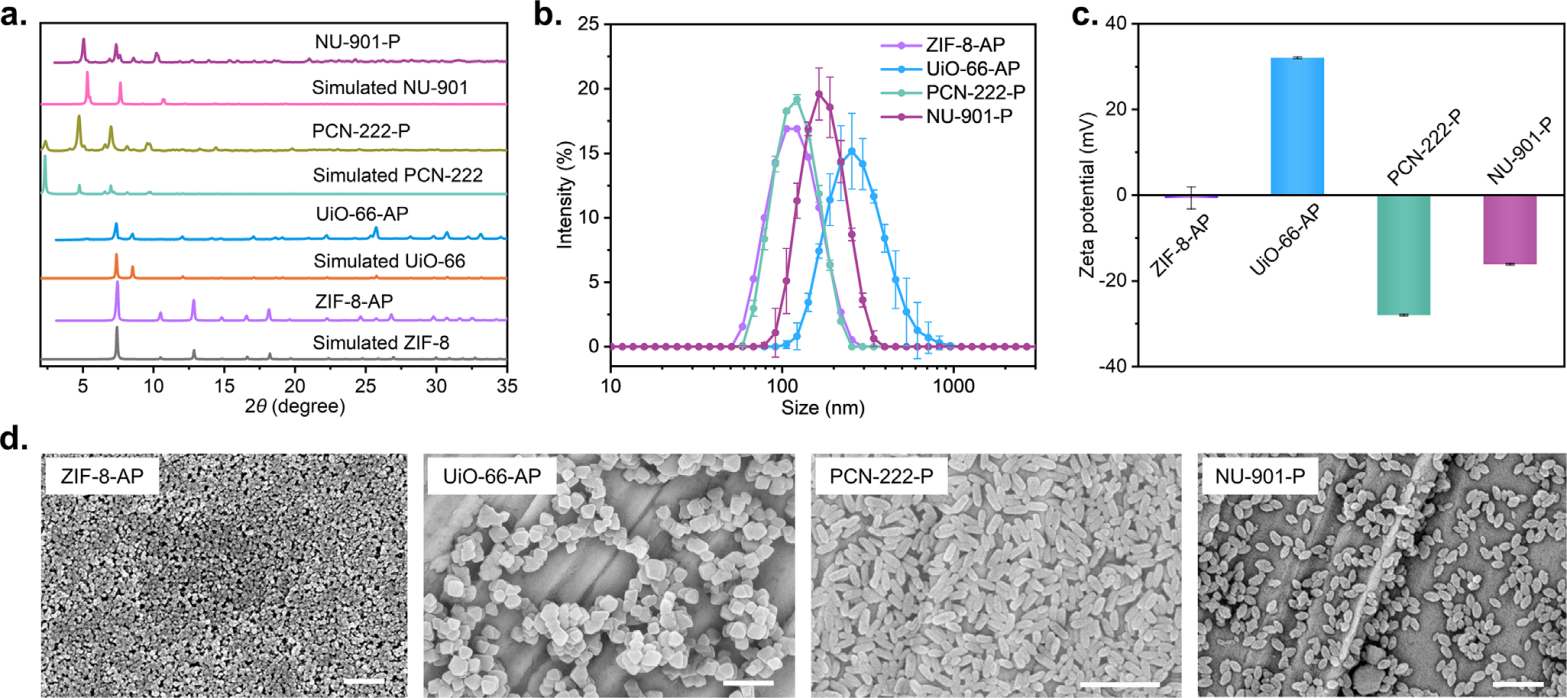
Characterization of ZIF‐8‐AP, UiO‐66‐AP, PCN‐222‐P, and NU‐901‐P. a) Simulated and experimental PXRD patterns. b) Intensity‐average diameter of water suspension of PEGylated MOFs, *n* = 3 for UiO‐66‐AP, PCN‐222‐P, and NU‐901‐P; *n* = 1 for ZIF‐8‐AP. c) Zeta potential of water suspensions of PEGylated MOFs, *n* = 3. d) SEM images of PEGylated MOFs. Scale bar, 500 nm.

Following the in silico screening, we synthesized the three Zr‐based MOFs – NU‐901 (isoreticular to NU‐1000), PCN‐222, and UiO‐66 – alongside the Zn‐based ZIF‐8 as model systems, owing to their demonstrated utility in drug delivery.^[^ ^13^, ^16^, ^26^, ^46^ ^]^ There are structural distinctions between these frameworks, with UiO‐66 and ZIF‐8 being microporous (i.e., < 2 nm pores) and NU‐901 and PCN‐222 being mesoporous. Moreover, the pyrene‐based linker in NU‐901 (H_4_TBAPy) and porphyrin‐based linker in PCN‐222 (tetrakis (4‐carboxyphenyl)porphyrin, TCPP) exhibit intrinsic fluorescence, and the MOFs themselves are capable of macromolecular encapsulation.^[^ ^49^ ^]^ To enhance physiological stability, all MOFs except ZIF‐8 were functionalized with methoxy PEG‐phosphate (PEG, Mn = 5 k) following a previously reported protocol,^[^ ^17^ ^]^ preserving the crystallinity post‐modification, as confirmed by PXRD (**Figure** **2**a). ZIF‐8 was PEGylated using PEG‐NH_2_. To enable fluorescent tracking, we relied on linker emission for PEGylated NU‐901 and PCN‐222 (denoted NU‐901‐P and PCN‐222‐P, respectively), whereas for UiO‐66 and ZIF‐8, prior to PEGylation, we coprecipitated the fluorescent dye Alexa Fluor AF647 (denoted UiO‐66‐AP and ZIF‐8‐AP, respectively). Figure 2 shows the characterization of the formulated MOFs. Figure 2b illustrates the particle sizes obtained through dynamic light scattering (DLS) in water, while Figure 2d provides the scanning electron microscopy (SEM) images; **Table** **1** summarizes the particle sizes, polydispersity index (PdI), and zeta potential (ζ‐potential). SEM particle sizes range from 46 to 138 nm, whereas effective hydrodynamic diameters measured by DLS range from 110 to 274 nm. All formulations maintained sub‐2 µm hydrodynamic diameters, in principle, enabling phagocytosis and cellular uptake. The PdI values range from 0.058 to 0.23, with PCN‐222‐P and NU‐901‐P being monodisperse (PdI < 0.1). Surface charge analysis showed that PCN‐222‐P and NU‐901‐P exhibit negative ζ‐potential after PEGylation (Table 1 and Figure 2c), whereas ZIF‐8‐AP has a neutral surface, and UiO‐66‐AP shows a positive ζ‐potential value of +32 mV, likely due to dye interactions.

**Table 1:** Characterization of _nano_MOFs. SEM and DLS particle size, polydispersity, and z‐potential.

MOF	Particle size [nm]^a)^	Hydrodynamic diameter [nm]^b)^	PdI	Z‐potential [mV]
ZIF‐8‐AP	45.8 ± 15.1	110 ± 0.0	0.17	−0.83
UiO‐66‐AP	138.0 ± 26.5	274.1 ± 5.0	0.23	32.0
PCN‐222‐P	117.9 ± 22.0	115.4 ± 2.1	0.058	−28.2
NU‐901‐P	128.2 ± 20.4	213.0 ± 3.1	0.068	−16.3

^a)^ Measured by SEM – errors represent a standard deviation of 100 particles;

^b)^ Measured by DLS in water.

## 4 Peripheral Blood Mononuclear Cell Viability

**Figure 3:**
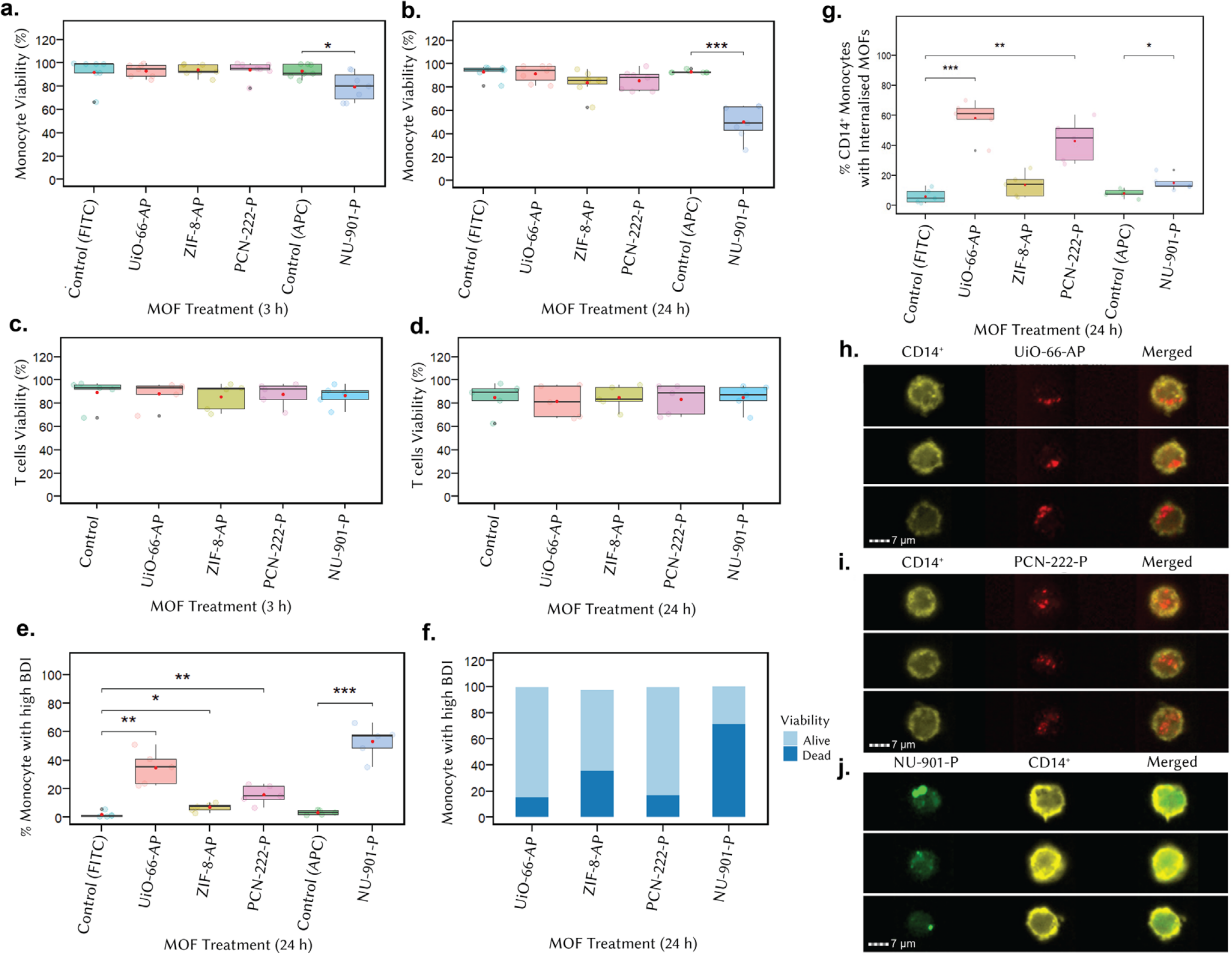
Ex vivo analysis of biocompatibility and interaction of _nano_MOFs with monocytes and T cells within PBMC. a,b) Viability of CD14^+^ monocytes and c,d) CD3^+^ T cells after treatment with MOFs for 3 and 24 h measured by flow cytometry, respectively. e) Percentage of CD14^+^ monocytes with high BDI MOF fluorescence after MOF treatment, as measured by imaging flow cytometry. f) Alive/dead monocyte percentages present within the high BDI MOF gates shown in (e) for different MOF candidates plotted in stacked bars. Light blue bars indicate the percentage of CD14^+^ BDI high double positive cells within the live gate, and the dark blue bars indicating CD14^+^ BDI high double positive cells within the dead gate. The alive/dead populations are gated using viability staining. g) Percentage of CD14^+^ monocytes with internalized BDI MOFs signals, as measured by imaging flow cytometry. h) Single and merged example images of CD14^+^ (yellow) cells residing within the UiO‐66‐AP (red) internalization gates. Similarly, example images of CD14^+^ cells are shown in (i). PCN‐222‐P in red and (j). NU‐901‐P in green from within internalization gates. Statistical analysis was carried out using *t*‐test in RStudio (*n* = 5; *** *p* ≤ 0.001, ** *p* ≤ 0.01, * *p* ≤ 0.05).

We evaluated MOF compatibility with human peripheral blood mononuclear cell (PBMC) populations using parallel conventional and imaging flow cytometry analysis to compare internalization kinetics of the _nano_MOFs in mixed cell populations present in blood. CD14^+^ monocytes (phagocytic) and CD3^+^ T lymphocytes (non‐phagocytic) were analyzed after 3 and 24 h of incubation with the four PEGylated _nano_MOFs in tissue culture media at a concentration of 10 µg mL^−1^. Post‐incubation and staining with CD3/CD14 fluorescently conjugated cell surface marker antibodies and a viability stain, we evaluated _nano_MOF toxicity in PBMC by examining changes in CD14^+^ monocytes (**Figure** **3**a,b) and CD3^+^ T cells viability (Figure 3c,d) using conventional flow cytometry. Cells were plotted based on size (forward scattered light, FSC) and granularity (side scattered light, SSC) parameters to enable live monocyte and lymphocyte gating, followed by gating according to their CD14^+^ and CD3^+^ positive signals and viability staining (**Figure** S1, Supporting Information). Figure 3a,b shows the viability of monocytes upon treatment with _nano_MOFs at 3 and 24 h, respectively. We observed no evidence of toxicity for UiO‐66‐AP, ZIF‐8‐AP, and PCN‐222‐P. However, NU‐901‐P significantly reduced CD14^+^ monocyte viability at 3 h (*p* ≤ 0.05) and 24 h (*p* ≤ 0.001). Flow cytometry histogram plots are shown in **Figure** S2 (Supporting Information). This effect is evident as early as 3 h, with an additional shift observed between 3 and 24 h, indicating a progressive loss of viability over time. The increase in cells positive for the dying cell marker during NU‐901‐P incubation supports a non‐acute decrease in viability over time. Although MOFs generally exhibit good chemical and thermal stability, they can degrade in biological media due to phosphate attacks.^[^ ^17^ ^]^ For NU‐901, this degradation releases its pyrene derivative linker, H_4_TBAPy, which may contribute to its cytotoxicity as the MOF interacts with monocytes over time – an observation which we first noted by examining physicochemical descriptors correlated to the toxicity classification of H_4_TBAPy by our ML‐pipeline.^[^ ^32^ ^]^

CD3^+^ T cell viability remained unaffected across all _nano_MOFs (Figure 3c,d), consistent with their non‐phagocytic nature. The selective monocyte toxicity suggests phagocytosis‐driven _nano_MOF accumulation via endocytosis, where active NU‐901‐P internalization concentrates toxic degradation byproducts and overwhelms cellular clearance mechanisms. This selective cytotoxicity is particularly insightful when considered in the context of the comparative design of this study. Both NU‐901‐P and the non‐toxic PCN‐222‐P are constructed from the same Zr‐based metal center and feature similar node connectivity, strongly suggesting that the observed toxicity is attributable to the H_4_TBAPy linker, a derivative of pyrene.

## 5 Association and Internalization of MOFs in PBMCs

Conventional flow cytometry provides precise phenotypic information, cell counts, and viability assessment of MOF‐cell interactions but lacks imaging capabilities.^[^ ^50^, ^51^ ^]^ Imaging flow cytometry overcomes this limitation by combining microscopy with flow cytometry, enabling the quantification of fluorescence at different cellular localizations.^[^ ^52^, ^53^ ^]^ To investigate the effects of MOF on monocytes, we employed both techniques. Figures S3 and S4 (Supporting Information) outline the gating strategies for CD14^+^ monocytes and CD3^+^ T cells. Bright detail intensity (BDI) analysis measured punctuate, high‐intensity signals derived from MOF associated with CD14^+^ staining cells (Figure 3e). Gated BDI‐high MOF^+^ cells were assessed for viability (Figure S3, Supporting Information), revealing variable MOF association: NU‐901‐P and UiO‐66‐AP showed higher interaction than PCN‐222‐P and ZIF‐8‐AP (Figure 3c). NU‐901‐P was predominantly associated with dead or dying monocytes (Figure 3f). Pearson product‐moment correlation analysis confirmed a strong negative relationship between NU‐901‐P uptake and monocyte viability (Table S1, Supporting Information) likely due to the release of its cytotoxic pyrene‐derivative linker. UiO‐66‐AP exhibited high monocyte affinity – likely due to its positively charged surface^[^ ^15^, ^54^, ^55^, ^56^ ^]^ – but minimal cytotoxicity, whereas ZIF‐8‐AP and PCN‐222‐P showed lower association and no toxicity. These findings highlight that MOF toxicity is driven by internalization of cytotoxic components rather than by their association alone.

Imaging flow cytometry further allowed the quantification of MOF internalization (Figures S3–S5, Supporting Information). By distinguishing internal and membrane‐associated MOFs using CD14^+^ or CD3^+^ cell surface markers,^[^ ^57^ ^]^ we determined internalization levels (Figure 3g–j). Figure 3g shows the percentage of live CD14^+^ gated monocytes exhibiting particle internalization; Figure 3h–j show example images of CD14^+^ cells for each MOF, except for NU‐901‐P, because this parallel analysis strategy only analyzed viable cells for internalization and NU‐901‐P was highly toxic to cells. All remaining internalized MOFs presented as bright, small punctate clusters – expected when accumulating within endo/lysosomal compartments of CD14^+^ monocytes (Figure 3h–j). Endo/lysosomal compartments are the primary sites for processing internalized materials – including MOFs – and their localization suggests that MOFs are processed within monocytes, potentially influencing their biological activity and effects. These findings align with previous observations that particles, including MOFs, are directed to CD107a‐positive endo/lysosomal compartments in monocytic cells and tissue culture cell lines.^[^ ^54^, ^58^ ^]^ It is also worth noting that a higher percentage of cells displayed internalized UiO‐66‐AP (*p* ≤ 0.001) compared to PCN‐222‐P (*p* ≤ 0.01) (Figure 3g), suggesting an enhanced route of uptake for UiO‐66‐AP particles by monocytes, again, due to its positively charged surface, as has been previously described for other professional phagocytes^[^ ^15^, ^54^, ^55^, ^56^ ^]^ Using imaging flow cytometry, we observed only a small but relevant amount of NU‐901‐P being internalized into live monocytes (Figure 3g), despite the high number of cells associating with NU‐901‐P (BDI high) (Figure 3e). This can be explained by the high percentage of NU‐901‐P^+^ cells within the dead CD14^+^ population, as the internalization analysis was performed only on live CD14^+^ cells and not on those appearing in the dead cell gate. This may again suggest a time‐dependent factor in the toxicity of NU‐901‐P, with the toxic effect taking place once the MOF tracks to endo/lysosomal compartments, where it would be broken down in the acidic lysosomes. Brightfield images show that monocytes incubated with NU‐901‐P exhibit morphological differences with clear signs of blebbing (Figure S5, Supporting Information), indicative of toxicity and the initiation of cell death.

Analysis of CD3^+^ T cells (Figure S6a, Supporting Information) showed no significant internalization of UiO‐66‐AP, ZIF‐8‐AP, or PCN‐222‐P, consistent with their non‐phagocytic nature. However, NU‐901‐P exhibited notable T‐cell association 24 h post‐incubation, likely through exterior membrane attachment and subsequent uptake via dynamic membrane recycling.^[^ ^59^ ^]^ Figure S6c,d (Supporting Information) shows examples of NU‐901‐P associating with T cells, either internally or membrane‐associated. To distinguish between particles that were truly internalized and those attached to the T cell surface, we analyzed cells with high NU‐901‐P BDI signals using the CD3 membrane marker as the cell boundary (Figure S6b, Supporting Information). Here, “internal to the CD3 membrane marker” refers to MOF particles located inside the T cell, within the cytoplasm, and clearly enclosed by the boundary defined by the CD3^+^ membrane marker. In contrast, “CD3 membrane marker‐associated” indicates MOF particles that are attached to the outer surface of the cell membrane but not internalized.

We observed that NU‐901‐P predominantly remained associated with the CD3^+^ membrane and was not internalized (Figure S6b, Supporting Information). Internalized NU‐901‐P appeared in CD3^+^ cells predominantly as small, singlet puncta, which are much smaller than those observed in monocytes, with some observed in fainter clusters residing in the cytoplasm of T cells (Figure S6c, Supporting Information). In contrast, non‐internalized NU‐901‐P formed large, bright puncta attached to the T cell membrane (Figure S6d, Supporting Information). T‐cell membrane attachment has previously been reported for other small metal‐based particles.^[^ ^60^ ^]^ Furthermore, NU‐901 loaded with the fluorescent dye calcein has previously been reported to enter HeLa cells predominantly via caveolae‐mediated and clathrin‐mediated pathways.^[^ ^5^ ^]^

## 6 Cytokine Response and Inflammation

**Figure 4:**
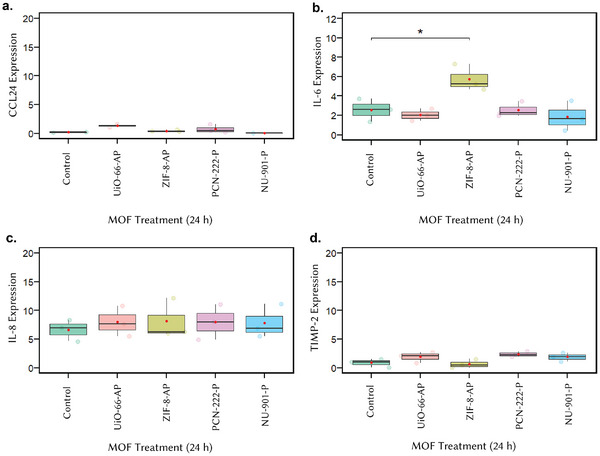
Semi‐quantitative analysis on cytokines. a) CCL24, b) IL‐6, c) IL‐8, and d) TIMP‐2 expression level in PBMC cell suspension (Y‐axis: arbitrary units (a.u.)). Statistical significance was analyzed using *t*‐test in RStudio, with statistical significance established at *p* ≤ 0.05 (*).

To assess the immunotoxic effects of our _nano_MOF candidates, we analyzed cytokine expression in PBMC samples, which are biomarkers for signs of immunotoxicity. Using the Inflammation Protein Array C1 (RayBiotech), we screened for 20 major inflammatory cytokines after 24 h of MOF exposure. Figure S7 (Supporting Information) provides an example of the cytokine array, while **Figure** **4** presents a semi‐quantitative analysis of CCL24 (Figure 4a), IL‐6 (Figure 4b), IL‐8 (CXCL8) (Figure 4c), and TIMP‐2 (Figure 4d), the cytokines detected in all samples at varying intensities. Among these, only IL‐6 showed a significant increase, exclusively in response to ZIF‐8‐AP treatment (Figure 4b). IL‐6 is a pleiotropic cytokine involved in early‐stage inflammation, modulating innate and adaptive immune responses and playing a role in cytokine release syndrome.^[^ ^61^, ^62^, ^63^ ^]^ The pro‐inflammatory signature of ZIF‐8, characterized by the specific release of IL‐6, is a critical finding that flags it as unsuitable for passive drug delivery applications pursuant to our screening pipeline. Mechanistically, this response is complex, as the degradation of the ZIF‐8 framework in acidic endosomes releases both Zn^2+^ and the 2‐MI linker. While ZIF‐8 is widely studied for its biocompatibility, Zn^2+^ ions released from ZIF‐8 have been linked to cytotoxicity in murine macrophages.^[^ ^64^ ^]^ Moreover, studies have reported that free Zn^2+^ can be immunomodulatory and induce IL‐6 production in certain cell types.^[^ ^65^ ^]^ Additionally, zinc oxide nanoparticles have been shown to induce IL‐6 release in dendritic cells.^[^ ^66^ ^]^ Though structurally distinct from ZnO, the potential for Zn^2+^ release from ZIF‐8 in biological media may similarly trigger an immune response. However, a more specific mechanism has been identified for the imidazole component. Recent literature has demonstrated that the imidazole linker acts as an agonist for endosomal TLRs^[^ ^46^ ^]^ – a well‐established pathway for inducing IL‐6, as discussed previously. Although our study was not designed to definitively decouple the relative contributions of the metal ion versus the organic linker, the specific TLR‐agonist activity of the imidazole provides a strong mechanistic hypothesis for the observed pro‐inflammatory effect. Ultimately, from the perspective of our screening pipeline, the key takeaway is the presence of a specific immune response – which, irrespective of its precise origin – is sufficient to disqualify ZIF‐8 as a candidate for stealth, passive drug delivery. Despite the low affinity of ZIF‐8‐AP for CD14^+^ cells and minimal internalization, the specific PBMC subpopulations responsible for IL‐6 production remain unclear. However, given the lack of cell‐type‐specific signatures observed,^[^ ^67^ ^]^ it is very likely the IL‐6 response represents an inflammatory response produced by professional phagocytes upon uptake and subsequent toxicity, as observed by us (Figure 3e,f), and others in professional phagocytic cell types.^[^ ^64^, ^66^ ^]^ Our overall findings with regards to the observed immune response of ZIF‐8 align with previous reports from Jaklenac, Langer et al.,^[^ ^46^ ^]^ and Horcajada et al.^[^ ^30^ ^]^

## 7 In Vivo Immune Response

**Figure 5:**
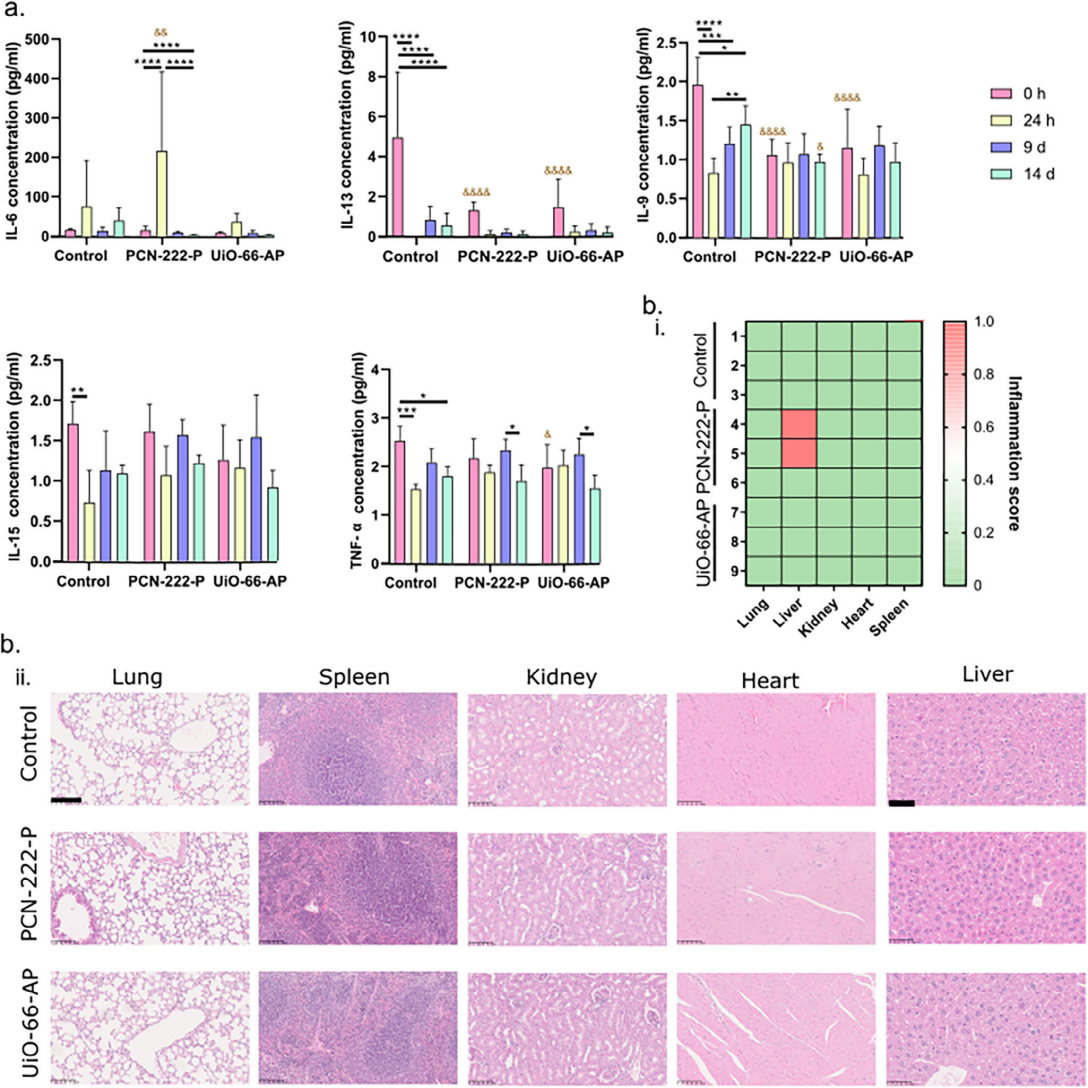
Screening of inflammatory cytokines in mouse plasma upon MOF treatment. a) Quantitative analysis of IL‐6, IL‐13, IL‐9 IL‐15, and TNF‐α at different time points upon NPs administration. Mean ± SD, & *p* < 0.05 vs control at selected timepoint, && *p* < 0.01 vs control at selected timepoint, &&& *p* < 0.001 vs control at selected timepoint, &&&& *p* < 0.0001 vs control at selected timepoint, * *p* < 0.05, ** *p* < 0.01, *** *p* < 0.001, and **** *p* < 0.0001, ordinary ANOVA test with Tukey's multiple comparisons test. b) Histopathology of the collected mouse organs, including lungs, liver, kidney, heart, and spleen: i) heatmap showing the inflammation scores of the Control group (mice 1, 2, and 3), PCN‐222‐P (mice 4, 5, and 6), and UiO‐66‐AP (mice 7, 8, and 9). The values (scores) assigned for each of these lesions were as follows: 0 (no change) when no injury or the observed changes were within the normal range, and 1 (minimum) when changes were sparse but exceeded those considered normal. ii) histopathological images stained with H&E of lung, spleen, kidney, and heart. Scale bar: 100 µm and 50 µm (Liver images).

Following ex vivo screening, we evaluated the immunogenicity of UiO‐66‐AP and PCN‐222‐P using an in vivo model. ZIF‐8‐AP and NU‐901‐P did not progress to in vivo analysis due to issues flagged during the in silico and ex vivo screening. Although the cytotoxicity of NU‐901‐P is problematic, the pro‐inflammatory nature of ZIF‐8 renders it unsuitable for passive drug delivery. This is not to say that ZIF‐8 is without biomedical applications. Indeed, in instances where provoking an immune response is desirable, the adjuvant‐like properties of ZIF‐8 may be useful, as demonstrated in previous studies.^[^ ^46^, ^68^, ^69^ ^]^ However, given our study's scope for screening “stealth” carriers that do not elicit immune responses, only UiO‐66‐AP and PCN‐222‐P progressed to the in vivo stage. SKH1 hairless mice were intravenously administered a single dose of UiO‐66‐AP and PCN‐222‐P via retro‐orbital injection (7.5 mg mL^−1^, 100 µL per mouse). Mouse plasma was collected at 0‐, 1‐, 9‐, and 14‐days post‐administration. As shown in **Figure** **5**a, cytokine levels, including IL‐6, exhibited a transient increase within 24 h post‐administration across all treatment groups, returning to baseline by day 14, consistent with the immune profile observed in the control group (PBS). Figure S8 (Supporting Information) compares the analyte concentrations in mouse plasma upon MOF treatment for all cytokines studied. Besides IL‐6, other cytokines, including IL‐13, IL‐9, IL‐15, and TNF‐α, did not show a consistent or marked increase compared to the control group, suggesting a lack of robust immune activation. These data imply that the immune system did not exhibit a broad inflammatory response to the PEGylated _nano_MOFs, as evidenced by the relatively small cytokine variations observed for these markers (Figures S8–S10, Supporting Information).

Histopathological analysis of organ tissues (Figure 5b), including the liver, lungs, kidneys, heart, and spleen, further supported these findings, revealing minimal inflammation (Figure 5b‐i). Notably, while PCN‐222‐P exhibited slight Kupffer cell activation – indicative of minor hepatic stress – no significant damage or pathological changes were observed in other tissues. These observations suggest that both UiO‐66‐AP and PCN‐222‐P are well tolerated by the immune system, causing minimal acute immune activation or inflammatory damage. The slight activation of Kupffer cells in the liver (PCN‐222‐P) could reflect mild transient immune responses that did not lead to significant pathology (Figure 5b‐ii).

The absence of a strong immune response across the treatment groups, along with the favourable biodistribution profiles of both UiO‐66‐AP and PCN‐222‐P, suggests that these materials exhibit favourable biocompatibility and minimal immunotoxicity. This aligns with previous ex vivo and computational predictions regarding their safety, supporting their potential as promising candidates for biomedical applications. Additionally, we administered PCN‐222‐P to healthy animals to assess the toxicity of the MOFs. On Days 0, 1, and 14 following retro‐orbital administration of PCN‐222‐P, the animals were euthanized. The collected organs were then digested to quantify the Zr levels via ICP analysis (Figure S11, Supporting Information). Following PCN‐222‐P administration, the MOFs accumulated primarily in the lungs, with levels decreasing over time, reaching 5 ppm by day 14. At 14 days post‐administration, Zr levels were minimal, with ≈4 ppm in the liver, 0.02 ppm in the heart, 0.1–29 ppm in the spleen, and 0.63 ppm in the kidneys. Overall, these results underscore the importance of assessing cytokine responses, nanoparticle clearance, and histopathology as part of a comprehensive immunotoxicity evaluation of MOFs.

## 8 Outlook

Our integrated assessment of formulated _nano_MOFs reveals framework‐dependent immune interactions. NU‐901‐P exhibited pronounced CD14^+^ monocyte toxicity within 24 h of incubation linked to phagocytosis‐driven degradation and linker release. Although significant amounts of UiO‐66‐AP and PCN‐222‐P were internalized by monocytes, this did not translate to changes in viability. CD3^+^ T cells remained unaffected by _nano_MOF treatment, despite the unusual internalization of NU‐901‐P due to endocytic recycling adhering to the T‐cell membrane. This controlled comparison between the three Zr‐based MOFs provides compelling evidence that the linker is the key source of toxicity. While all three frameworks share the same biocompatible Zr‐metal center, only NU‐901‐P induced significant cytotoxicity. This argument is further strengthened by a direct comparison between NU‐901 and PCN‐222, which not only share the same metal center but also have similar node connectivity. With these critical structural features being equal (*ceteris paribus*), the key differentiating variable is the chemical nature of the organic linker. The linker in NU‐901 is a derivative of pyrene, a PAH with well‐documented cytotoxic and immunotoxic properties, a hazard correctly identified by our initial in silico screening.

Upon screening a panel of major inflammatory cytokines, we observed an increase in IL‐6 expression in PBMCs treated with ZIF‐8‐AP. In vivo, upon intravenous administration of UiO‐66‐AP and PCN‐222‐P in SKH1 hairless mice, we observed a transient increase in IL‐6 levels 24 h post‐administration, which returned to baseline by day 14, consistent with the control group (PBS). In addition to IL‐6, we observed slight variations in other cytokines – IL‐13, IL‐9, and IL‐15 – though these differences did not indicate significant immune activation. Histopathological examination of the liver, lungs, kidneys, heart, and spleen showed minimal inflammation, with slight Kupffer cell activation in the PCN‐222‐P group, indicative of minor hepatic stress but no significant tissue damage. These in vivo findings align with ex vivo data, where the internalization of UiO‐66‐AP and PCN‐222‐P into monocytes did not result in major immune responses or pathological changes. While we acknowledge that certain stimuli can induce “trained immunity” (a form of innate immune memory) even after a single exposure, a definitive assessment of long‐term effects on either innate or adaptive memory would necessitate studies with repeated exposures, which was beyond the scope of this initial safety‐profiling work.^[^ ^70^, ^71^ ^]^

In conclusion, our study establishes a multi‐scale pipeline that bridges computational, ex vivo, and in vivo models to systematically de‐risk MOF development for clinical translation. Our approach moves beyond simplistic toxicity classification to generate a more nuanced understanding of “immune fingerprints” for each material. Our head‐to‐head comparison of clinically relevant MOFs identified candidates suitable for passive drug delivery (**Table** **2**). By identifying these toxicity determinants and distinguishing between cytotoxic, pro‐inflammatory, and inert profiles – we provide a roadmap for the rational design of MOF‐based therapies – matching a material's intrinsic properties to a specific therapeutic goal. This profiling will accelerate the translation of safer nanomedicines, though further studies should explore long‐term immune memory effects and tissue‐specific clearance pathways to build a complete safety profile for clinical use.

**Table 2:** Comparative summary of _nano_MOF immunotoxicity profiles and suitability for clinical translation.

	NU‐901‐P	PCN‐222‐P	UiO‐66‐AP	ZIF‐8‐AP
Composition	Zr, Pyrene‐derivative linker (H_4_TBAPy)	Zr, Porphyrin linker (TCPP)	Zr, Carboxylate linker (BDC)	Zn, Imidazole linker (2‐MI)
In silico prediction	Toxic linker	Safe	Safe	Toxic metal & linker
Porosity>	Mesoporous	Mesoporous	Microporous	Microporous
Ex vivo monocyte viability	Significant decrease	No change	No change	No change
Ex vivo cytokine response	No significant change	No significant change	No significant change	Significant IL‐6 increase
In vivo outcome (mice)	Not advanced	Minimal inflammation, Transient cytokine increase	Minimal inflammation, Transient cytokine increase	Not advanced
Translational suitability	Low (Intrinsic Cytotoxicity)	High (Biocompatible)	High (Biocompatible)	Low (Pro‐inflammatory)
Potential application	Niche chemo‐immunotherapy	Passive drug delivery	Passive drug delivery	Vaccine adjuvant/ Immunotherapy

## 9 Experimental Section

### In Silico Screening

The screening was performed using ML models previously reported by us.^[^ ^32^ ^]^ For visualizing the chemical landscape (Figure 1c), the t‐SNE method^[^ ^72^ ^]^ was used, since it preserves local similarity in high‐dimensional space in a lower‐dimensional map. A total 197 physicochemical descriptors were calculated at different hierarchical levels using the rdkit library.^[^ ^73^ ^]^ Univariate statistical tests based on the ANOVA F‐value were then used to select 110 features most correlated to the toxicity – which were used as input for t‐SNE. For complete details, the reader may refer to the previous work.^[^ ^32^ ^]^

### Materials

All reagents (unless otherwise stated) were purchased from Sigma Aldrich and used without further purification. AFDye 647 DBCO was purchased from Click Chemistry Tools. For the synthesis, Milli‐Q water (18.2 MΩ.cm resistivity at 25 °C) was used. The Zr_6_ cluster was synthesized according to a reported procedure.^[^ ^74^ ^]^ Methoxy PEG phosphate was prepared according to a reported method.^[^ ^75^ ^]^

### Synthesis of UiO‐66‐AP

*p*‐azidomethyl benzoic acid‐modified UiO‐66 was synthesized according to a reported procedure.^[^ ^76^ ^]^ To the aqueous suspension of *p*‐azidomethyl benzoic acid‐modified UiO‐66 (10 mg in 3 mL H_2_O), Alexa Fluor™ 647 DBCO (5 mg, 4.4 µmol) in 0.2 mL H_2_O was added. The mixture was stirred for 3 days at room temperature. Afterward, the reactant was centrifuged and washed with H_2_O three times (14 700 rpm, 20 min), followed by re‐suspension of the nanoparticle in H_2_O. Then, an aqueous solution of methoxy PEG phosphate (50 mg, Mw = 5000) was added. After stirring at room temperature for 16 h, the reaction mixture was centrifuged to remove the unreacted methoxy PEG phosphate (3 × 14 700 rpm for 20 min). The final product was redispersed in H_2_O for characterization and analysis.

### Synthesis of ZIF‐8‐AP

1 mg of Alexa Fluor™ 647 NHS Ester was added to an aqueous solution of 2‐methylimidazole (3.15 mmol, 0.5 mL) through hand shaking and then subjected to ultrasound for 30 min. Next, an aqueous solution of Zn(CH_3_COO)_2_·2H_2_O (30 mg, 0.5 mL) and Tween 80 (0.1 mg, 10 µL) was gently mixed with the above mixture. The resulting mixture turned turbid after 15 s and was left undisturbed at room temperature for 5 min. The resulting ZIF‐8 nanoparticles were centrifuged at 9500 rpm for 10 min, washed with ethanol twice, and finally mixed and stirred with a 0.3% PEG‐NH_2_ (w/w) aqueous solution for 30 min. The mixture was then centrifuged at 9500 rpm for 10 min. The collected wet pellets were finally redispersed in DI water. The as‐prepared ZIF‐8‐AP nanoparticles were stored at 4 °C for future use.

### Synthesis of PCN‐222‐P

Tetrakis (4‐carboxyphenyl)porphyrin (22.5 mg, 28.5 µmol), Zr_6_ cluster (38 mg, 14.2 µmol), 8 mL DMF, and 130 µL of trifluoroacetic acid were ultrasonically dissolved in a 20 mL threaded vial. The resultant mixture was placed in the 120 °C block heater for 5 h. After cooling to room temperature, the obtained purple sample was collected by high‐speed centrifugation (15 000 rpm, 35 min), followed by washing with fresh DMF three times and exchanging with ethanol three times. The final product was re‐dispersed in H_2_O for further use. Methoxy PEG phosphate (50 mg, Mw = 5000) solution (10 mL, 25 mg mL^−1^ in H_2_O) was added to the aqueous suspension of PCN‐222 (5 mL, 10 mg mL^−1^). After stirring at room temperature for 16 h, the reaction mixture was centrifuged and washed with fresh H_2_O three times (14 700 rpm, 20 min). The final product was redispersed in H_2_O and stored at 4 °C for biological assays.

### Synthesis of NU‐901‐P

1,3,6,8‐Tetrakis(p‐benzoic acid)pyrene (H_4_TBAPy) (25 mg, 40.4 µmol), ZrOCl_2_·8H_2_O (121 mg, 375.5 µmol), 4‐aminobenzoic acid (400 mg, 2.92 mmol), trifluoroacetic acid (200 µL), and DMF (20 mL) were ultrasonically dissolved in a 50 mL threaded vial. The mixture was heated at 140 °C for 50 min, whereupon a yellow suspension formed. After cooling to room temperature, the obtained sample was collected by centrifugation (4700 rpm, 35 min), followed by washing with fresh DMF three times and exchanging with ethanol three times. The final product was re‐dispersed in H_2_O. Methoxy PEG phosphate (50 mg, Mw = 5000) solution (10 mL, 25 mg mL^−1^ in H_2_O) was added to the aqueous suspension of NU‐901 (5 mL, 10 mg mL^−1^). After stirring at room temperature for 16 h, the reaction mixture was centrifuged and washed with fresh H_2_O three times (14 700 rpm, 20 min). The final product was redispersed in H_2_O and stored at 4 °C for biological assays.

### Characterization

PXRD data were collected on a Bruker D8 DAVINCI diffractometer at 298 K using Cu Kα radiation. The calculated PXRD patterns were produced using the Mercury program and single‐crystal reflection data. For SEM analysis, the samples were coated with Au for 40 s and imaged using a FEI Nova Nano SEM 450. For DLS, measurements were recorded on a Zetasizer Nano ZS (Malvern Instrument Ltd., U.K.) equipped with a He−Ne laser operating at 633 nm at 25 °C. Data are representative of three replicate measurements. Error bars represent the standard deviation of three measurements.

### Cell Culture and Treatment

Experiments using human PBMC were performed under the approval of the UK NHS Health Research Authority, Edgbaston Research Ethics Committee, REC reference 18/WM/0221. Human PBMCs were isolated from leukocyte cones purchased from the National Blood Service (Cambridge, UK) as described previously.^[^ ^60^ ^]^ Prior to experimentation, PBMCs were thawed, washed, and rested in RPMI cell culture medium supplemented with 2 mM L‐glutamine (Thermo Fisher Scientific, 21875034) and 10% foetal calf serum (Thermo Fisher Scientific, 11550356) for 2 h. The cells were counted and resuspended in culture medium at the final concentration of 1 × 10^6^ cells mL^−1^ in FACS tubes. 10 µg of MOFs in solution were then added to the cells and incubated for 3 h and 24 h at 37°C, 5% CO_2_. Following MOF incubation with PBMCs for 24 h, supernatants were collected and stored at −80 °C for cytokine production assay. After incubation with MOFs, PBMCs were washed with 2 mL of phosphate‐buffered saline (PBS, Thermo Fisher Scientific, 10010023) before staining with Live/Dead stain (Thermo Fisher Scientific, L34955) on ice for 20 min according to the manufacturer's protocol. The cells were then washed with FACS wash buffer (filtered PBS containing 1% bovine serum albumin and 0.01% of sodium azide). Cells were split into two groups for staining for either CD3‐PE, or CD14‐PE. All antibodies were purchased from BD Science and diluted according to the manufacturer's protocols. After incubation with antibodies on ice in the dark for 20 min, the cells were washed with FACS buffer, followed by fixation on ice with 2% paraformaldehyde (PFA) for immediate acquisition on the same day. Unstained and single‐stained compensation tubes, with and without MOF treatment, were also prepared for spectral overlap compensation.

### Conventional Flow Cytometry

The CyAn ADP 9‐colour analyser (Beckman Coulter, Ltd, High Wycombe, UK) was equipped with 405 nm, 488 nm, and 642 nm solid‐state lasers, with 11 detectors in standard configuration. Summit software was used for acquisition and analysis (Beckman Coulter). Samples were filtered through 35 µm nylon cell strainer mesh tubes (BD Biosciences), and 300,000 events were acquired for each sample. For data analysis, events were first plotted as forward versus side scatter (FS/SS) on a log scale, and debris was excluded before further analysis.

### Imaging Flow Cytometry

The ImageStreamX Mark I platform (Amnis) was used in standard configuration, equipped with 405 and 488 nm lasers for excitation and a 785 nm laser. INSPIRE (Amnis) and IDEAS software (Amnis, Seattle, WA, USA) were used for acquisition and analysis, respectively. The machine was fully calibrated and passed all tests prior to the acquisition. Single‐stain compensation tubes, as well as the MOFs (plus cells) only tube, were prepared alongside test samples. Samples were filtered through 35 µm nylon cell strainer mesh tubes prior to acquisition, and a minimum of 30 000 events per sample were acquired. Compensation matrices were generated by running single‐stained samples and analysed using IDEAS software. For analysis, the analysis wizard was used to gate cells in best focus, followed by gating single cells using a scatter plot of brightfield area versus aspect ratio. Next, CD3^+^/CD14^+^ PE‐positive cells were gated based on their fluorescent intensity. Similarly, to gate “alive” or “dead” cells, the intensity of the viability marker in Ch1 was used. To quantify the number of CD3^+^ or CD14^+^ cells positive for MOF fluorescence and identify the viability of MOF‐associated cells, the Bright Detail Intensity (BDI) feature was used to measure the intensity of bright spots caused by MOF signals within the cell area of each cell image. The intensity of bright spots within the cell image with a radius of 3 pixels or less was measured using the BDI R3 feature. For this analysis step, CD14^+^ or CD3^+^ gated cells were plotted for BDI R3 of the MOF channel signal (Ch02 or Ch05) versus the intensity of the phenotypic marker (CD3 or CD14). The BDI high gate was then drawn beyond the natural population cluster of intensity signals for the MOF channel based on the no‐MOF control samples. Cells within the BDI high gate were considered positive for MOF, plotted for dead cell stain intensity, and further gated as “alive” or “dead” according to the intensity of the viability staining signal to establish the viability of MOF‐associated cells. The measurement of internalization was based on the internalization feature, which calculates the ratio of intensity of the MOF signal measured inside a cell to the intensity of the MOF signal for the entire cell. A mask eroded by 4 pixels from the cell membrane staining (either CD14 or CD3) cell image was created, and the intensity of the MOF signal was measured within the eroded mask to calculate the internalization score. Internalization high (hi) gates were placed beyond the natural population background values from the negative samples without MOFs. A full “working example” of the gating and analysis strategies is shown in Figures S3 and S4 (Supporting Information).

### Cytokine Protein Microarray Analysis

An antibody‐based membrane array for measuring human inflammatory factors (RayBio® C‐Series Human Inflammation Antibody Array kit) was purchased from Raybiotech (Norcross, GA, USA). This approach was favorable as an unbiased and wide‐ranging initial screening of the presence or absence of key factors indicating an inflammatory response, rather than looking for individual cytokines. PBMCs/MOFs supernatant samples were thawed from −80°C fresh for each test, and the assay was performed according to the manufacturer's protocol. Each antibody was spotted in duplicate, and a total of 4 donor samples were analyzed. Complete blots of cytokine arrays were imaged using the G:Box Imaging System, and images were acquired using Image Studio (ver.5.2) software. Images were then analyzed in ImageJ to quantify the signal intensities for each spot. The changes in the signals were compared with those of the control patients and evaluated in RStudio.

### Animal Studies

All animal experiments were approved by the Ethical Committee and the Animal Welfare and Ethics Body of Nova Medical School (21_01_ORBEA.4) and followed the animal research guidelines of Nova Medical School and the Directorate‐General for Food and Veterinary Medicine. SKH1 Hairless Mice (6 weeks old, average weight = 20 g) were purchased from Charles River Laboratories (France) and acclimatized for 1 week before the experiments. Animals were kept under aseptic conditions with light/dark cycles of 12 h. Water and standard pellet food were provided ad libitum. The animal procedures were performed under isoflurane anesthesia (IsoFlo 100% p/p). Mice were randomized into three groups (15 animals, 5 per group): 1) control (PBS), 2) PCN‐222‐P, and 3) UiO‐66‐AP. Mice received 7.5 mg mL^−1^ via retro‐orbital injection, 100 µl per mouse (50 ul in each side). After administration, blood was collected at 0, 3 h, 24 h, 48 h, day 6, day 9, and day 14 and stored at −80 °C until further characterization. At the endpoint (day 14), mice were euthanized by cervical dislocation. Mouse plasma was analyzed using GeniePlex Mouse Inflammation 17‐Plex (AssayGenie, Ireland) as described by the manufacturer's protocol. The following analytes were quantified: IFN‐γ, IL‐1α, IL‐1β, IL‐6, IL‐9, IL‐10, IL‐12p70, IL‐13, IL‐15, IL‐2, IP‐10, KC, MCP‐1, MIP‐1α, MIP‐1β, RANTES, and TNF‐α. A dot plot was generated using visualization functions available in the ggplot2 library in R. Liver, spleen, pancreas, heart, and lungs were harvested and fixed in 4% formalin for histological analysis. The presence of epithelial damage in mouse organs was assessed by hematoxylin‐eosin (H&E) staining. Briefly, formalin‐fixed paraffin‐embedded (FFPE) samples were sectioned at 3 µm thickness, mounted on positively charged glass slides, and dried overnight at 65 °C. After deparaffinization, samples were rehydrated and stained with H&E. H&E staining was evaluated by a histological score, which was expressed in ordinal scale units. The grading system for microscopic examination of inflammatory response with scores: 0 (no change) when no injury or the observed changes were within normal range, and 1 (minimum) when changes were sparse but exceed those considered normal.

### Inductively Coupled Plasma‐Optical Emission Spectroscopy (ICP‐OES) Analysis

SKH1 Hairless Mice (6 weeks old, average weight = 20 g) were purchased from Charles River Laboratories (France) and acclimatized for 1 week before the experiments. Animals were kept under aseptic conditions with light/dark cycles of 12 h. Water and standard pellet food were provided ad libitum. The animal procedures were performed under isoflurane anesthesia (IsoFlo 100% p/p). Mice were randomized into two groups (10 animals, 5 per group): 1) control (PBS), 2) PCN‐222‐P. Mice received 7.5 mg mL^−1^ via retro‐orbital injection, 100 µl per mouse (50 ul in each side). After NP administration, mice were euthanized by cervical dislocation and the organs (liver, spleen, pancreas, heart, and lungs) were harvested. ICP‐OES analysis was performed using a Horiba Jobin–Yvon (Ultima, France). Samples were freeze‐dried, weighed, and dispersed in 1 mL of nitric acid and left to stand at RT in the fume cupboard overnight. After this time, the clear solution was obtained, which was then diluted with Millipore water and analyzed for Zr content by comparing it to the standard Zr solutions. Two animals (*n* = 2) per tissue were analyzed by ICP‐OES.

### Statistical Analysis

Results are reported as mean ± SD of 3–7 independent experiments, each with more than three samples. Statistical analyses were performed using a paired *t*‐test in RStudio when comparing two groups, and statistical significance was established when *p* < 0.05.

## Conflict of Interest

D.F.‐J. has commercial interests in Vector Bioscience, a spin‐out for the commercialization of MOFs in healthcare applications. J.C. is a co‐founder and shareholder of TargTex S.A. – Targeted therapeutics for Glioblastoma Multiforme. J.C. is a member of the Global Burden of Disease (GBD) consortium of the Institute for Health Metrics and Evaluation (IHME), University of Washington (US) and is in the Scientific Advisory Board of Vector Bioscience.

## Supporting information



Supporting Information

## Data Availability

The data that support the findings of this study are available from the corresponding author upon reasonable request.
