# Supplementary material for: Multiscale Profiling of Nanoscale Metal‐Organic Framework Biocompatibility and Immune Interactions
Source: Adv Healthc Mater. 2025 Aug 7;14(29):e01809. doi: 10.1002/adhm.202501809 (PMC12616609; doi:10.1002/adhm.202501809)
Supplement: Supplementary file 1 — Supporting Information [file ADHM-14-0-s001.pdf]

ADVANCED HEALTHCARE MATERIALS

Supporting Information

for *Adv. Healthcare Mater.*, DOI 10.1002/adhm.202501809

Multiscale Profiling of Nanoscale Metal-Organic Framework Biocompatibility and Immune Interactions

Yunhui Zhuang, Bárbara B. Mendes, Dhruv Menon, Jhenifer Oliveira, Xu Chen, Fatma Demir Duman, João Conniot, Sergio Mercado, Xiewen Liu, Shi-Yuan Zhang, João Conde, Rachel E. Hewitt* and David Fairen-Jimenez**

Supporting Information for:

Multiscale profiling of nanoscale metal-organic framework immune interactions

Yunhui Zhuang[1], Bárbara B. Mendes[2], Dhruv Menon[1], Jhenifer Oliveira[2], Xu Chen[1], Fatma Demir Duman[1], João Conniot[2], Sergio Mercado[1], Xiewen Liu[1], Shi-Yuan Zhang[1], João Conde[2,], Rachel E. Hewitt[3,*] and David Fairen-Jimenez[1,*]*

[1]Adsorption & Advanced Materials Laboratory (AAML), Department of Chemical Engineering and Biotechnology, University of Cambridge, Philippa Fawcett Drive, Cambridge CB3 0AS, UK

[2]NOVA Medical School, Faculdade de Ciências Médicas, NMS, FCM, Universidade NOVA de Lisboa, Lisbon, Portugal

[3]Cellular Imaging and Analysis Facility, Department of Veterinary Medicine, University of Cambridge, Madingley Road, Cambridge CB3 0ES, UK

*e-mails: J.C.: joao.conde@nms.unl.pt; R.E.H., reh63@cam.ac.uk; D.F.-J., df334@cam.ac.uk

S1. Conventional Flow Cytometry Analysis

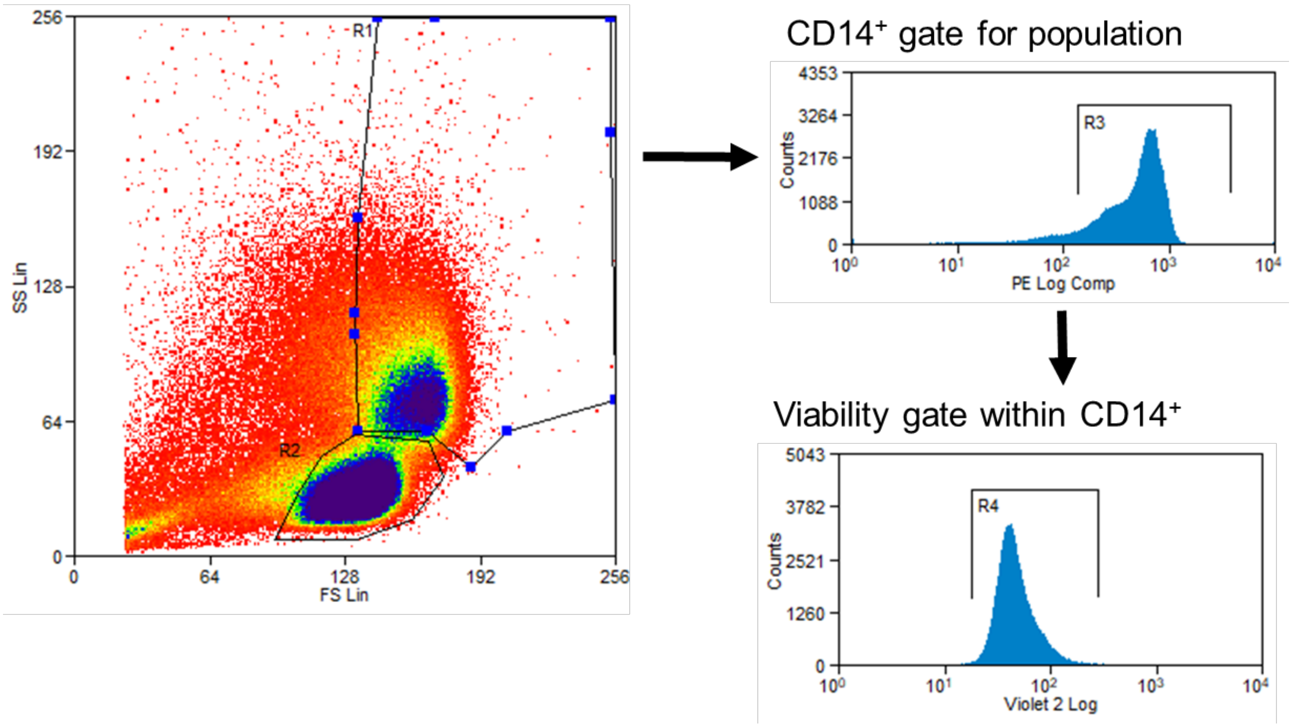

Supporting Fig. 1| Gating strategy for conventional flow cytometry analysis. Representative plots of PBMC from one donor. Cells were first plotted forward versus side scatter using side on a linear scale. A large gate was drawn to distinguish the monocytic (R1) and T cells (R2) populations within PBMC. The R1/R2 population was gated to the fluorescence intensity of phycoerythrin (PE) to identify $CD14^+$ or $CD3^+$ cells from the region. Following the identification of $CD14^+$ / $CD3^+$, gated cells were plotted against violet 2 fluorescent signals from their viability stain (R4). Note that PE fluorescence was used to detect and gate $CD14^+$ (monocytes) and $CD3^+$ (T cells) within the PBMC population.

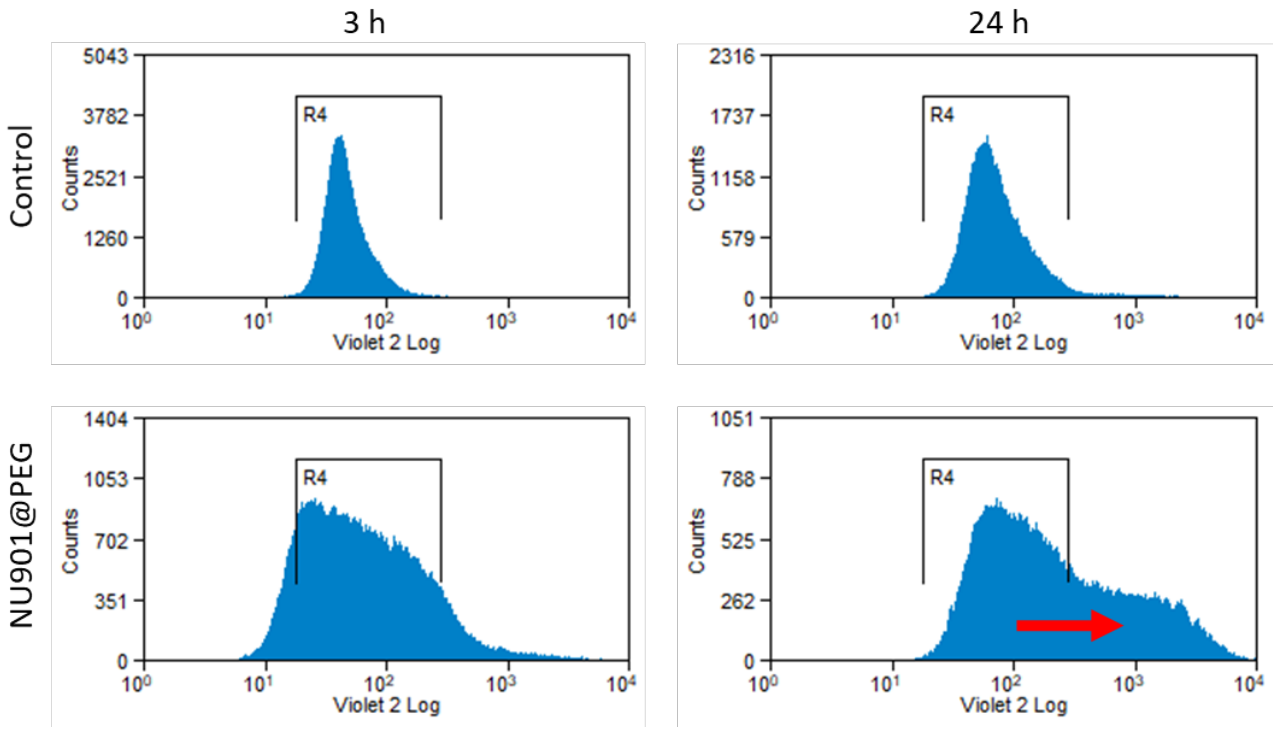

Supporting Fig. 2| CD14$^+$ monocytes viability shift for NU-901-P over 24 h of incubation. The viability gates for the control and PCN-222-P are shown as histogram plots. PCN-222-P viability stain shifted its peaks to the right of the x-axis, as indicated by the red arrow, which was related to a reduction in CD14$^+$ monocyte viability.

S2. Imaging Flow Cytometry Analysis

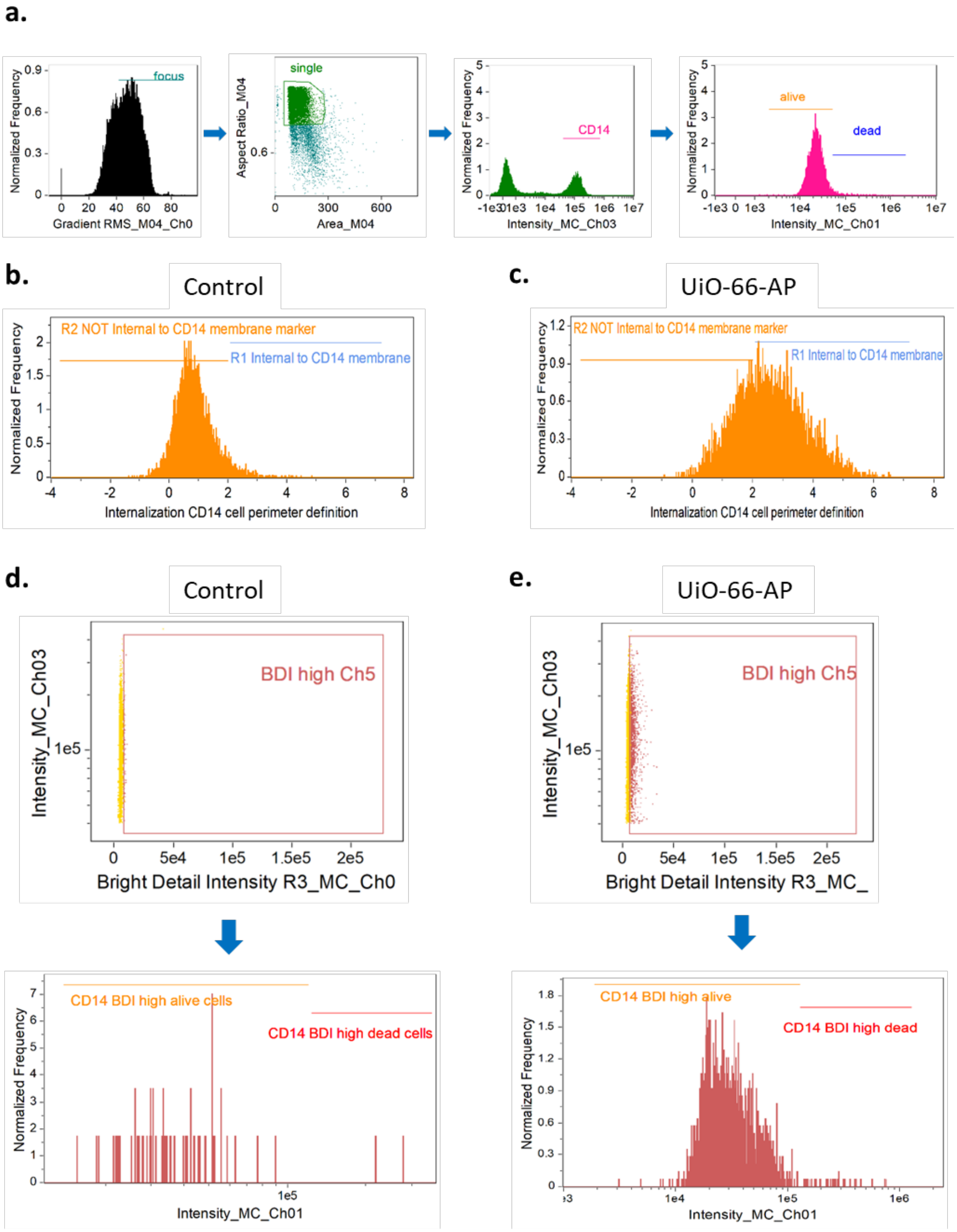

Supporting Fig. 3| Representative imaging flow cytometry analysis plots for monocytes from J1 donor. **a**, For all basic IFC analyses, the gradient root mean square (RMS) was used to gate cells in the best focus. RMS measures the overall sharpness of an image by calculating the root mean square of the intensity changes (gradients) between adjacent pixels. In imaging flow cytometry, cells that are in the best focus are identified and gated, ensuring that subsequent analyses are conducted on high-quality, well-focused images. Following this, the area versus aspect ratio was used to identify a single cell population of interest, followed by gating on fluorescence positives for CD14$^+$ (pink) in ch03 and alive (orange) / dead (blue) in ch01. **b** and **c**, Advanced analysis of control and UiO-66-AP, respectively. Histogram plots of internalization scores were used to create internalization high and low gates for alive CD14$^+$ cells. In addition, BDI Intensity feature R3 was used to gate CD14$^+$ cells (**d**) and (**e**), which were then further gated with fluorescent intensity from ch01 viability stain to identify whether the CD14$^+$ BDI high cells were alive or dead.

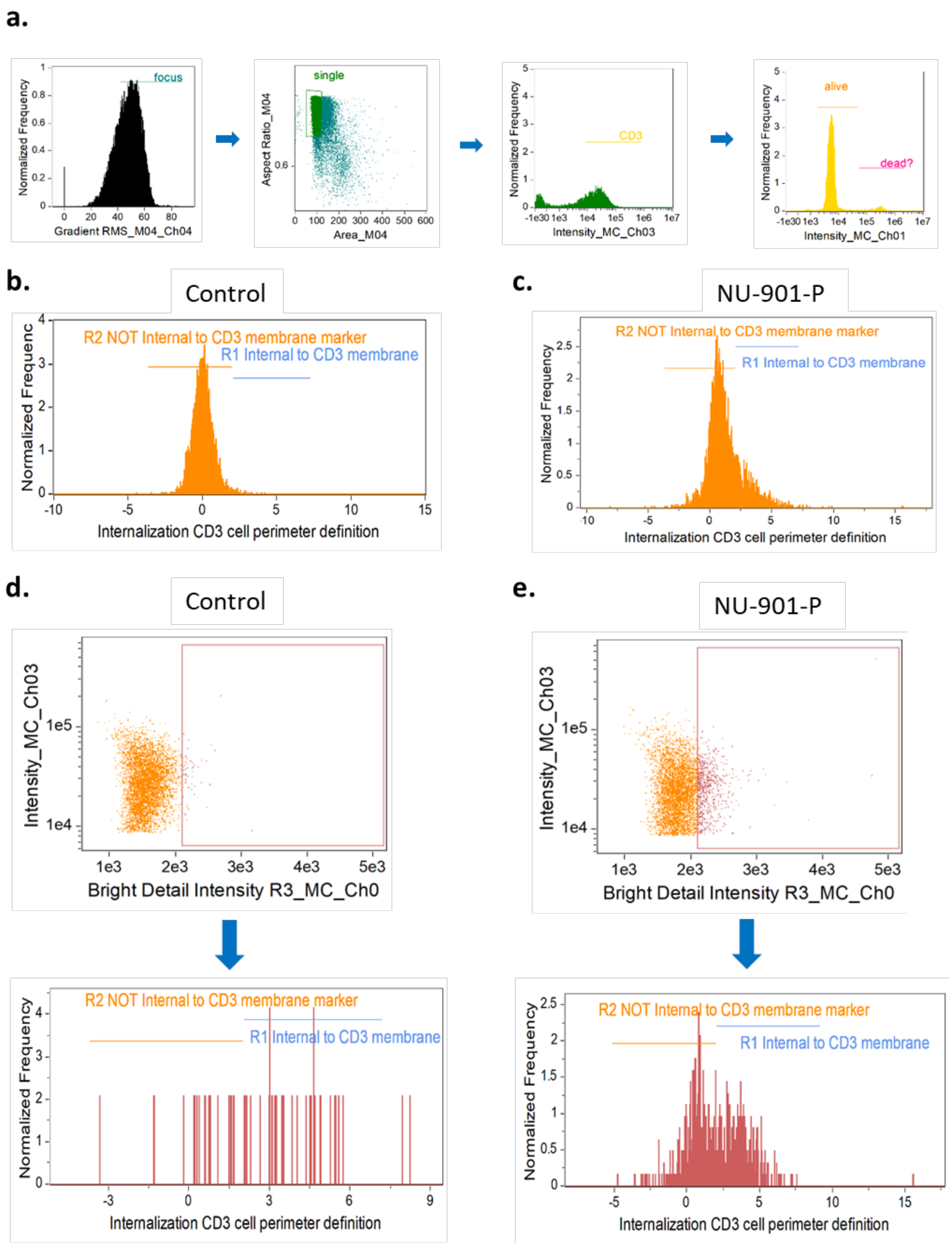

Supporting Fig. 4| Representative IFC analysis plots for T cells from the J1 donor. **a,** For all basic analysis (**a**), the gradient RMS was used to gate cells in the best focus. The area versus aspect ratio was used to identify the single-cell population, followed by gating on fluorescence positives for $CD3^+$ (pink) in ch03, and alive (orange)/dead (blue) in ch01. **b** and **c**, Advanced analysis of internalization for control and NU-901-P, respectively. Histogram plots of internalization scores were used to create internalization high and low gates for alive $CD3^+$ cells. Independently, BDI Intensity feature R3 was used to gate $CD3^+$ cells (**d**) and (**e**), respectively. These were then further gated with the internalization mask on the CD3 membrane marker to identify whether the $CD3^+$ BDI high cells were truly internalized into T cells.

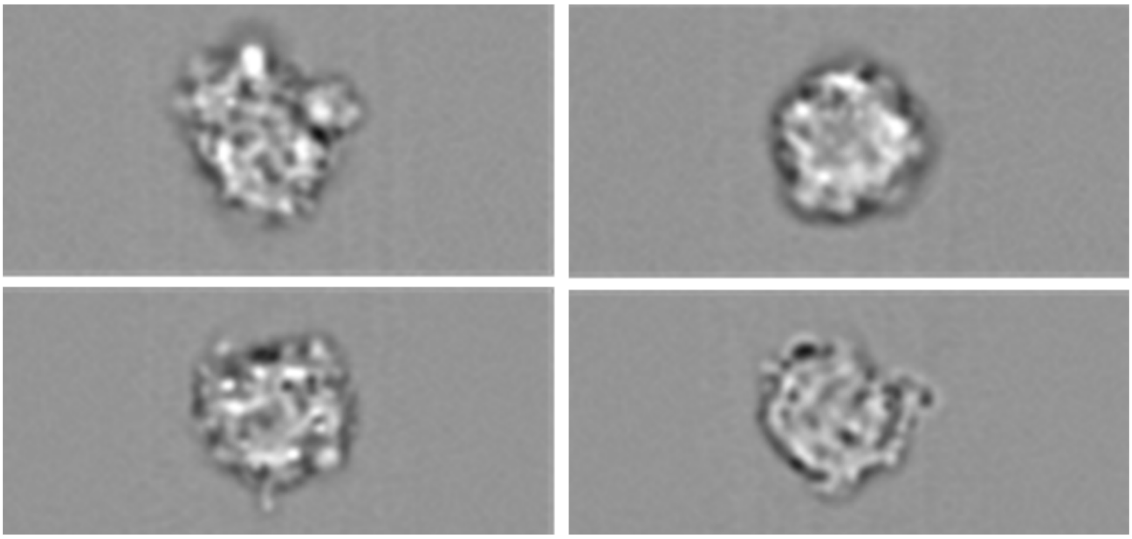

Supporting Fig. 5| Brightfield images of $CD14^+$ cells treated with NU-901-P.

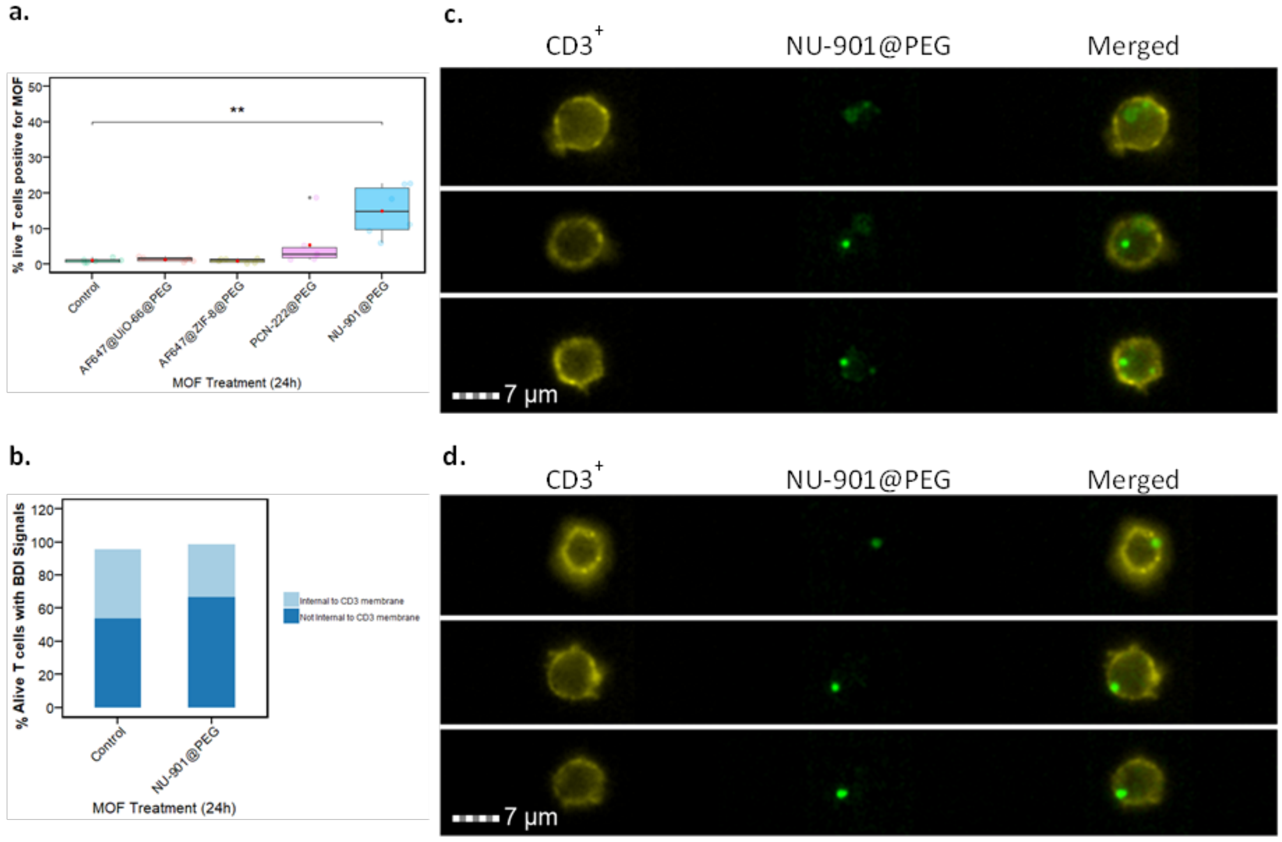

Supporting Fig. 6| a. % of live T cells positive for MOF. **b.** % of alive T cells with BDI signals for NU-901-P internal and not internal to CD3 membrane. **c.** Single and merged examples of $CD3^+$ cells with internalized NU-901-P. **d.** Single and merged examples of $CD3^+$ cells with NU-901-P attached to membrane.

S3. Cytokine Protein Microarray Analysis

a.

POS	POS	NEG	NEG	CCL11	CCL24	GCSF	GM-CSF
POS	POS	NEG	NEG	CCL11	CCL24	GCSF	GM-CSF
IFN-γ	IL-1α	IL-1β	IL-2	IL-3	IL-4	IL-6	IL-7
IFN-γ	IL-1α	IL-1β	IL-2	IL-3	IL-4	IL-6	IL-7
IL-8 (CXCL8)	IL-10	IL-11	IL12 p40	IL12 p70	IL-13	CCL1	TIMP-2
IL-8 (CXCL8)	IL-10	IL-11	IL12 p40	IL12 p70	IL-13	CCL1	TIMP-2
blank	blank	blank	blank	blank	blank	NEG	POS
blank	blank	blank	blank	blank	blank	NEG	POS

b.

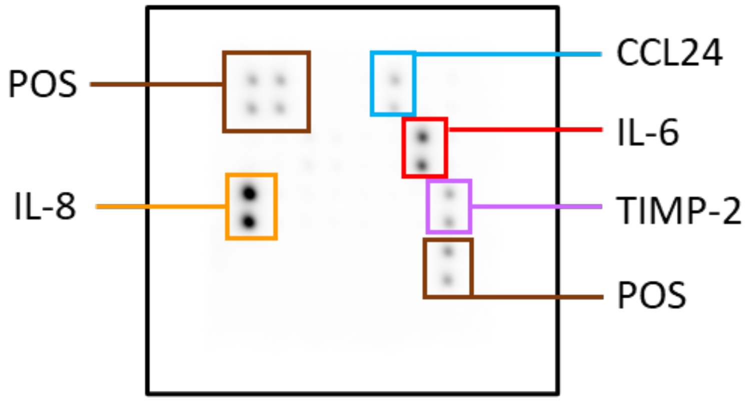

Supporting Fig. 7| Array map of RayBio® C-Series Human Inflammation Array C1. (**a**.) Array map provided by industry manufacturer [1]. (**b.**) Representative image of the protein array membrane after incubation with the cellular suspension from the control sample. Positive signals on the membrane are highlighted in different colors: positive control (POS), brown squares; CCL24, blue; IL-6, red; IL-8, orange; and TIMP-2, purple.

S4. Table of Pearson Product-Moment Correlation test results

Table S1 | Pearson Product-Moment Correlation Tests Results between % of alive CD14$^+$ cells and % of CD14$^+$ cells with BDIhi. All tests were performed in R Studio with independent samples n = 5.

MOF	Correlation Coefficient	Confidence interval at 95%	t-test	p-value of t-test	Statistical significance
AF647@UiO-66@PEG	-0.805	-0.989, 0.184	-2.353	0.1001	n/a
AF647@ZIF-8@PEG	0.141	-0.846, 0.910	0.247	0.8206	n/a
PCN-222@PEG	-0.834	-0.987, 0.266	-2.613	0.0795	n/a
NU-901@PEG	-0.944	-0.996, -0.366	-4.938	0.0159	*

S5. In vivo data

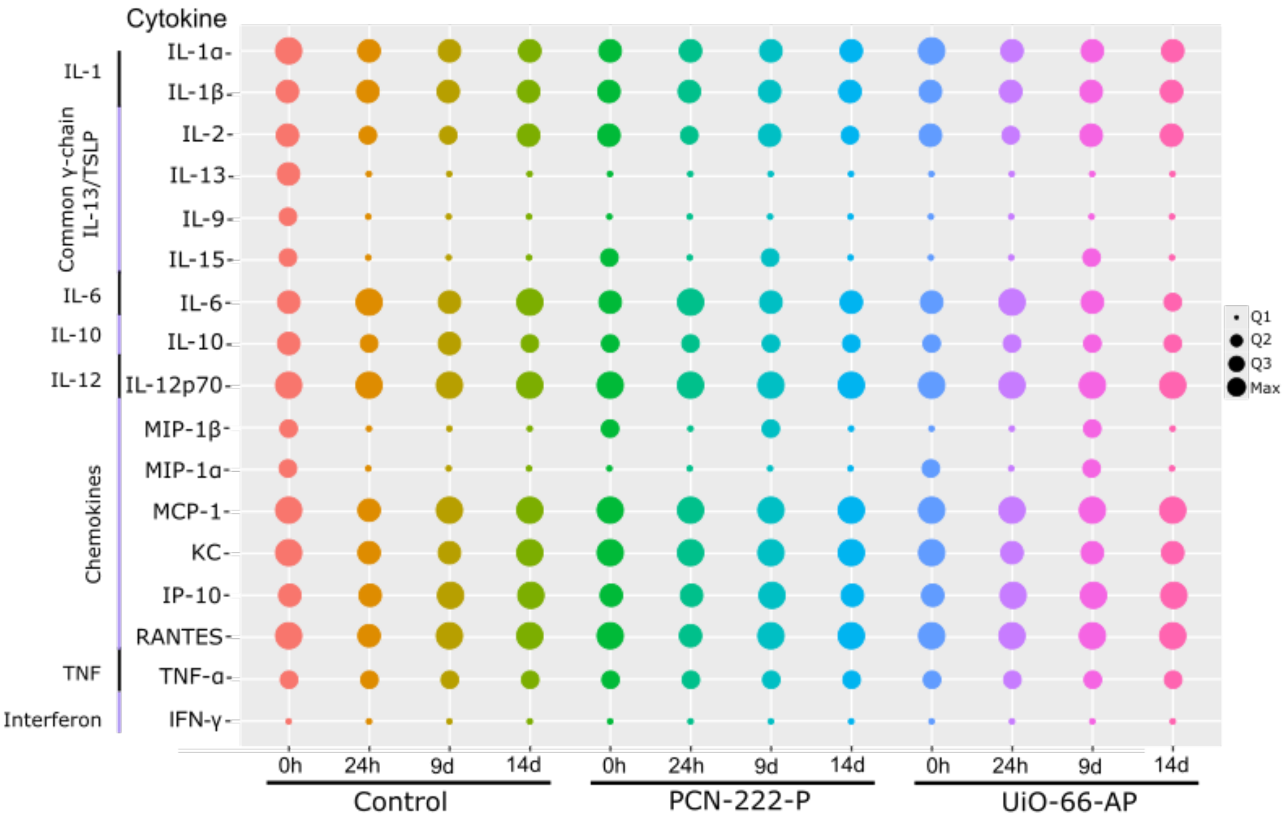

Supporting Fig. 8| Quantitative representation of analyte concentration (pg/mL) in mouse plasma upon MOF treatment for 0 h, 24 h, 9 d, and 14 d (Q1 = 1.2; Q2 = 3.7; Q3 = 16.7 and Max = 218), Supporting Fig. S9–S11. The variation in dot size reflects differences in the magnitude of immune activation or cytokine secretion in response to MOFs.

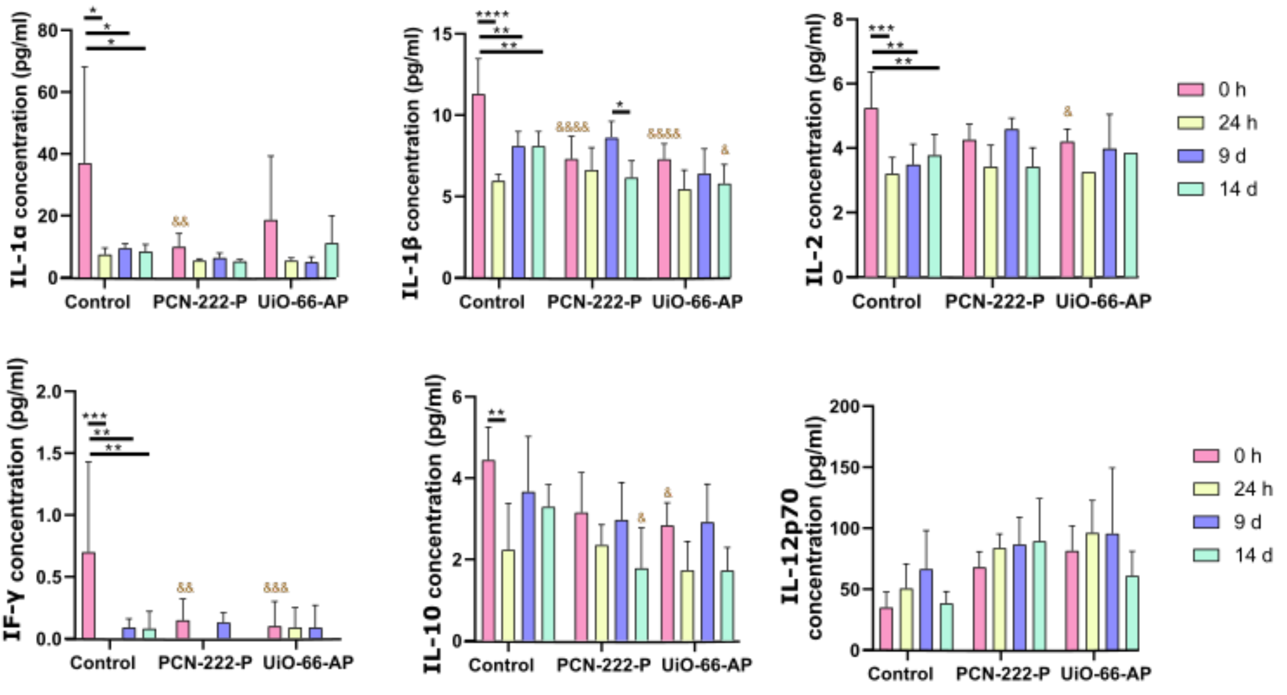

Supporting Fig. 9| Quantitative analysis of cytokines at different time points after nanoparticle administration. Mean ± SD, & $p < 0.05$ vs control at selected timepoint, && $p < 0.01$ vs control at selected timepoint, &&& $p < 0.001$ vs control at selected timepoint, &&&& $p < 0.0001$ vs control at selected timepoint, * $p < 0.05$, ** $p < 0.01$, *** $p < 0.001$, and **** $p < 0.0001$, ordinary ANOVA test with Tukey's multiple comparisons test.

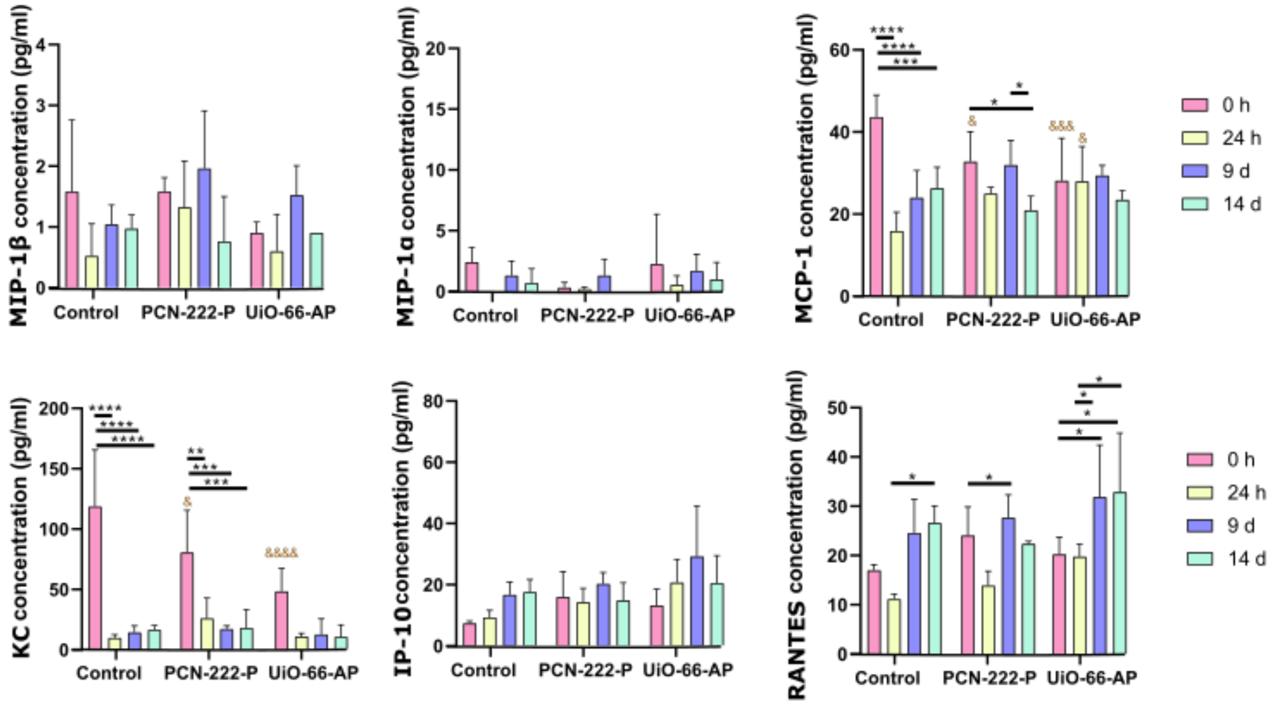

Supporting Fig. 10| Quantitative analysis of cytokines at different time points after nanoparticle administration. Mean ± SD, & $p < 0.05$ vs control at selected timepoint, && $p < 0.01$ vs control at selected timepoint, &&& $p < 0.001$ vs control at selected timepoint, &&&& $p < 0.0001$ vs control at selected timepoint, * $p < 0.05$, ** $p < 0.01$, *** $p < 0.001$, and **** $p < 0.0001$, ordinary ANOVA test with Tukey's multiple comparisons test.

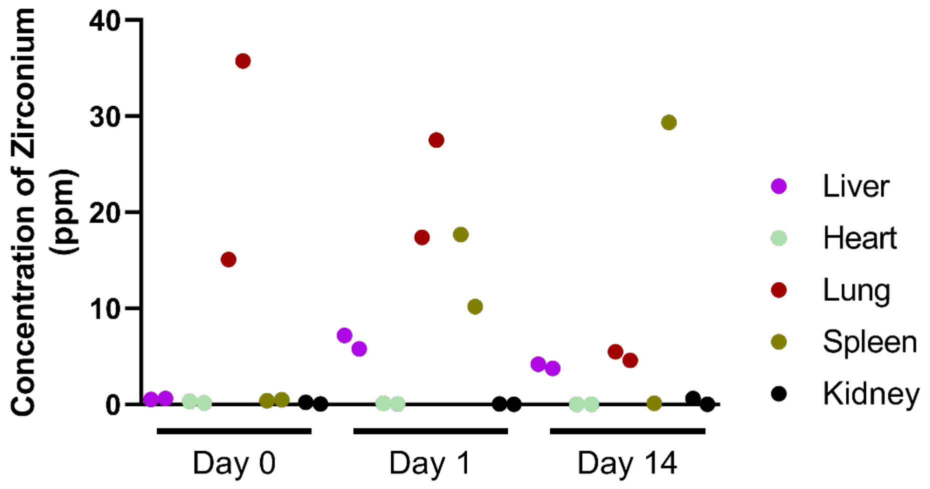

Supporting Fig. 11| Quantification of PCN-222-P in the mouse organs (Liver, Heart, Lung, Spleen and Kidney) by ICP analysis on days 0, 1, and 14 following nanoparticle administration (n = 2 animals).

S6. References

(1) Descotes, J. Translational Immunologic Safety Evaluation: A Perspective. *J. Immunotoxicol.* **2013**, *10* (1), 83–89. https://doi.org/10.3109/1547691X.2012.712067.
